# Women's experiences of living with chronic pain: A qualitative meta‐synthesis

**DOI:** 10.1111/bjhp.70023

**Published:** 2025-09-22

**Authors:** Sukhvinder Biring, Amy E. Burton, Lynn Dunwoody, Peter Kevern

**Affiliations:** ^1^ Centre for Health and Development, School of Health, Education, Policing and Sciences Staffordshire University Stoke‐on‐Trent UK; ^2^ Centre for Health and Development, School of Health, Education, Policing and Sciences Staffordshire University Stoke‐on‐Trent UK; ^3^ School of Psychology Ulster University Coleraine UK

**Keywords:** chronic pain, gender, meta‐synthesis, qualitative, women

## Abstract

**Objectives:**

The prevalence of chronic pain varies between males and females, and they also have distinct pain experiences. Improved understanding of these unique experiences is needed to improve support.

**Design:**

This meta‐synthesis aimed to develop a comprehensive understanding of women's lived experiences with chronic pain.

**Methods:**

Six electronic databases were searched in May and June 2022: PubMed Central, the Cumulative Index to Nursing and Allied Health Literature (CINAHL Plus), the Health Research Premium Collection, ScienceDirect, Web of Science and PsycINFO. Studies were included if they were full‐text journal articles, reported in English, presented qualitative findings obtained using qualitative research methods and focused on the experience of females over eighteen years old, living with chronic pain (not associated with cancer or conditions that are terminal). The search was updated in November 2024.

**Results:**

Analysis of the seventy studies retrieved identified four themes: Pain and Multiple Responsibilities; Countless Losses (and Their Psychological Effects); Lack of Understanding: Delegitimizing and Disempowering Encounters; and Solace and Self‐Empowerment. Confidence in all four themes was evaluated as high.

**Conclusions:**

These findings indicate that there are common themes that run through the lives of women living with chronic pain across a range of different age groups, locations and conditions. These domains present actionable opportunities to enhance pain management and well‐being for women living with chronic pain.

**Statement of contribution**

**What is already known?**

- Women are more likely than men to experience chronic pain.
- Women's experience of health care when living with chronic pain differs from the experience of men.
- Psychological factors play a key role in how chronic pain is experienced.

**What does this study add?**

- A comprehensive review and meta‐synthesis of the published qualitative evidence around women's experiences of chronic pain.
- An illustration of common themes and experiences relating to the challenges of multiple responsibilities, loss, lack of understanding and routes to empowerment.
- Evidence‐based recommendations for improving support through validation of pain experiences, increasing awareness, tailoring interventions and providing compassionate care.

## INTRODUCTION

Chronic pain is defined as pain that persists for longer than three months despite medication or treatment (NHS Inform, 2022). It is a complex phenomenon that can be experienced even in the absence of tissue damage or any other pathophysiological cause(s) (Crofford, 2015; International Association for the Study of Pain [IASP], 2020). One hundred million people are estimated to be living with chronic pain in Europe, which significantly impacts their quality of life (Community Research and Development Information Service, 2020). These figures are likely to increase in line with an ageing population and due to increased reporting of musculoskeletal (MSK) pain associated with long COVID (Geddes, 2021; Khoja et al., 2022).

As chronic pain is a multi‐dimensional phenomenon, biopsychosocial factors impact how individuals experience living with persistent pain (Adams et al., 2006; Love‐Jones, 2019). One of these factors is the sex of an individual, which is a key variable in differentiating illness prevalence and experiences: females are more likely to report or experience persistent pain than males (Mills et al., 2019), and they are more likely to experience high‐impact chronic pain (severe, disabling pain) than males of a similar age (Versus Arthritis, 2021). They also experience more pain‐related conditions, report higher levels of pain, longer durations of pain and experience it occurring more frequently (Fillingim et al., 2009; Fillingim & Maixner, 1995; Hallin, 2003; Keogh et al., 2005; Koons et al., 2018; Mills et al., 2019; Miyazaki & Yamamoto, 2009; Pieretti et al., 2016; Unruh, 1996).

There are also differences between how men and women experience medical care, and women are treated differently when it comes to pain (Lyman, 2021). They are more likely to be prescribed sedatives and anti‐anxiety medications than pain medications (Billock, 2018; Calderone, 1990; Koons et al., 2018), and they experience ill health and disability for a significantly greater period of their lives (Department of Health and Social Care, 2021).

It is estimated that seventy percent of individuals impacted by chronic pain are women (Kiesel, 2017). The IASP (2021) states that ‘Every day millions of women around the world suffer from chronic pain but many remain untreated’. This underscores the urgent need for targeted research on women living with chronic pain to better understand their specific needs, the challenges they face and to use these insights to improve outcomes and address care inequalities.

Furthermore, since numerous psychosocial factors play a crucial role in shaping the experience of pain and significantly contribute to its variability, understanding the key factors affecting women is essential. Addressing these factors could lead to improved health outcomes, reduced pain and better pain management (Cleveland Clinic, 2022; Engel, 1977; Melzack & Wall, 1965, 1996). Factors such as stress, anxiety and an intense focus on pain can dramatically heighten its perception, while others, like optimism and relaxation, possess the potential to mitigate it (Ambron, 2022). Similarly, beliefs about pain and self‐efficacy (Bandura, 1982; Firth et al., 2019) can influence pain, depending on whether these beliefs foster resilience or exacerbate feelings of helplessness (Leventhal et al., 2016; Paterick et al., 2017; Petrie & Weinman, 2006). Thus, exploring these factors within the context of women's experiences can unveil crucial dimensions that may be exacerbating their pain. This in‐depth understanding is essential for developing a holistic approach to pain management. Meta‐synthesis of these findings is also crucial as it provides an opportunity to “give voice” to their collective concerns and needs (Bennion et al., 2012); paving the way for effective interventions that are sensitive to women's needs, grounded in their lived experiences and tailored accordingly.

To the best of the researchers' knowledge, no systematic qualitative synthesis has been conducted that exclusively explores women's experiences of living with chronic pain and one that is not restricted to a particular condition. Qualitative syntheses have been identified that focus on a specific condition and that explore the experiences of men and women (Crowe et al., 2017; MacNeela et al., 2015; Toye et al., 2013). However, as mentioned above, there is variation between these groups. Thus, this review aims to systematically analyse qualitative findings on women's experiences with chronic pain to gain a more thorough and nuanced understanding.

## METHODS

### Design

Meta‐synthesis involves the interpretive integration of qualitative findings (Sandelowski & Barroso, 2007) and is frequently used for reviewing findings from qualitative studies (Finfgeld, 2003; Sandelowski & Barroso, 2007). The approach allows common themes, comparisons and differences based on sex to be identified, providing deeper insights than would be gained from a single empirical study (Erwin et al., 2011). The review protocol was registered in the International Prospective Register of Systematic Reviews (registration number: CRD42022331582).

### Search strategy

Initial scoping of the literature and the SPIDER tool (Cooke et al., 2012) was used to formulate the search strategy and the research question guiding this meta‐synthesis, which is presented in Table 1: What are women's experiences of living with chronic pain? The following electronic databases were searched in May and June 2022 (from inception to the search date): PubMed Central, the Cumulative Index to Nursing and Allied Health Literature (CINAHL Plus), the Health Research Premium Collection, ScienceDirect, Web of Science and PsycINFO. Searches were conducted using Boolean operators to combine terms. Citation searches were also undertaken. An updated search was conducted in November 2024 in PubMed Central, CINAHL Plus, the Health Research Premium Collection, ScienceDirect and PsycINFO. The search string adapted for ScienceDirect was used to conduct the searches. A filter was applied to include journal articles published from the date of the last search (June 2022) to November 2024.

**TABLE 1:** SPIDER framework and search terms.

SPIDER (Cooke et al., 2012)	Research question: What are women's experiences of living with chronic pain?
Sample	Women living with chronic pain (non‐malignant/terminal conditions): (Women OR female OR Gender) and “NOT cancer”
Phenomenon	Chronic pain (pain which lasts for longer than three months): (“Chronic pain”)
Design	Any study using qualitative methods to uncover women's experiences of living with chronic pain: (Qualitative OR “mixed methods” OR interview* OR “focus groups” OR explor*)
Evaluation	Experiences/views (daily life, health care, pain management, physical, social, and psychological impact): (experience* OR impact OR Perception OR view* OR attitude*)
Research type	Qualitative/Mixed methods: (Qualitative OR “mixed methods” OR interview* OR “focus groups” OR explor*)

*Note*: All searches were restricted to abstracts, and some databases allowed further filtration, which was utilized (stated below): PsycINFO (filter applied to show all journals), CINAHL Plus (search restricted to journals), and the ScienceDirect search had to be adjusted to meet the search requirements: (women OR female OR gender) AND (“chronic pain”) AND (qualitative OR “mixed methods” OR interviews) AND (experiences) NOT cancer.

### Eligibility criteria

To be included in the review, studies had to: (1) be primary research using qualitative or mixed methods; (2) focus on the experiences of females (aged over eighteen years) living with chronic pain (excluding those with cancer and terminal conditions); and (3) be reported in the English language in journals. There was no restriction on geographical region or setting. Studies were excluded if they focused on male experiences only, focused on pain with a malignant cause, or were literature reviews.

### Selection process

**FIGURE 1:**
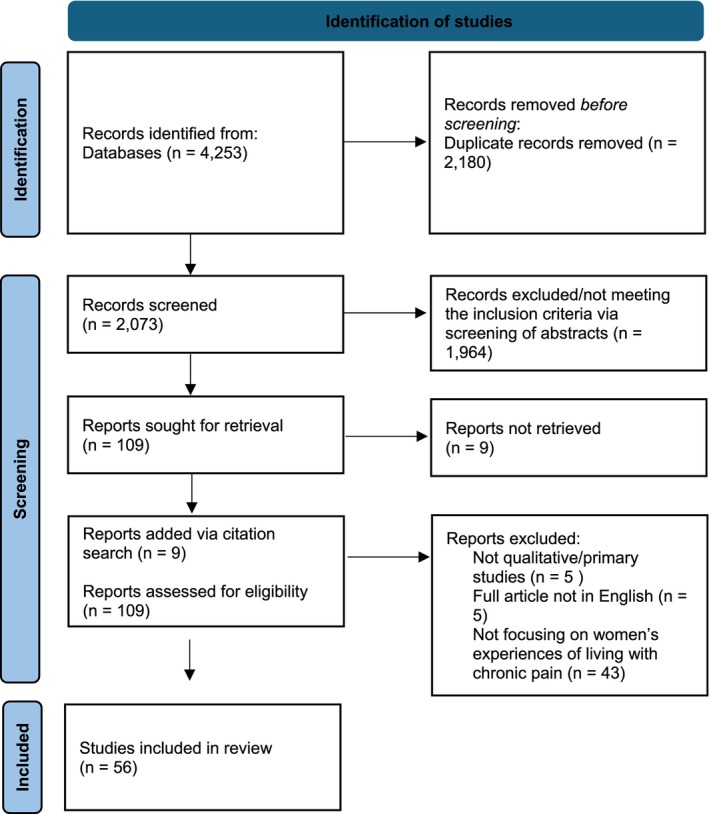
PRISMA flow diagram of study selection process. This diagram illustrates the process used to identify, screen and select studies included in the review. It shows the number of articles identified, included and excluded at each stage.

Zotero was used to record the search process and identify and remove duplicate articles. The first author screened all titles and abstracts against eligibility criteria and repeated this process with full texts. The Preferred Reporting Items for Systematic Reviews and Meta‐Analyses (PRISMA; Page et al., 2021) diagram (Figure 1) illustrates the number of articles identified, included and excluded and provides an overview of the study screening and selection process.

There were a total of 4253 records identified in the first search from the six database searches (Figure 1). Zotero was used to remove duplicates (*n* = 2180). The titles and abstracts of the remaining articles (*n* = 2073) were screened against the inclusion criteria. Articles not meeting the inclusion criteria were excluded (*n* = 1964). Of the 118 full articles sought for retrieval, 109 were retrieved and read in full, 56 studies were included in the review and 53 were excluded (not qualitative/primary studies: *n* = 5; full articles not in English*: n* = 5; studies not focusing on women's experiences of living with chronic pain: *n* = 43). In the updated search, 14 additional articles meeting the inclusion criteria were included from 306 results (excluding reviews, studies not focused on pain experiences or specifically on women's pain: *n* = 280 and duplicates: *n* = 12), resulting in a total of 70 studies included in the review (listed in Table 2).

**TABLE 2:** Numbered list of studies included in the review.

Ahlsen et al. (2014). (Un)doing gender in a rehabilitation context: A narrative analysis of gender and self in stories of chronic muscle pain. Allen et al. (2015). Exploring the experience of chronic pain among female Survival Sex Workers: a qualitative study Altun et al. (2023). Experiences of Assyrian refugee women seeking care for chronic pain: a qualitative study. Arman et al. (2020). Women's Lived Experiences of Chronic Pain: Faces of Gendered Suffering. Qualitative Health Research, 30(5), 772–782. Barnes et al. (2021). Exploring the emotional experiences of young women with chronic pain: The potential role of self‐compassion. Bostick et al. (2018). Pain Assessment Recommendations for Women, Made by Women: A Mixed Methods Study. Campbell et al. (2022). Women's experiences of navigating chronic pain within the context of living with an episodic disability. Campeau (2018). Adaptive frameworks of chronic pain: Daily remakings of pain and care at a Somali refugee women's health centre. Caton et al. (2024). “My Goal is…to get Through the Day Without Pain”: A Qualitative Study on Chronic Pain Experiences and Treatment Needs Among Child Caregiving Women. Dickson and Kim (2003). Reconstructing a meaning of pain: older Korean American women's experiences with the pain of osteoarthritis. Driscoll et al. (2018). Patient Experiences Navigating Chronic Pain Management in an Integrated Health Care System: A Qualitative Investigation of Women and Men. Dysvik et al. (2013). A narrative approach to explore grief experiences and treatment adherence in people with chronic pain after participation in a pain‐management program: A 6‐year follow‐up study. Evans and de Souza (2008). Dealing with chronic pain: Giving voice to the experiences of mothers with chronic pain and their children. Gonzalez et al. (2015). Life History of Women with Fibromyalgia: Beyond the Illness. Gullacksen and Lidbeck (2004). The life adjustment process in chronic pain: psychosocial assessment and clinical implications. Hallberg and Carlsson (1998). Psychosocial vulnerability and maintaining forces related to fibromyalgia. In‐depth interviews with twenty‐two female patients. Hallberg and Carlsson (2000). Coping with fibromyalgia: A qualitative study. Hervik et al. (2023). *Living with chronic headaches: A qualitative study from an outpatient pain clinic in Norway*. Horment‐Lara et al. (2022). “I don't want to be a burden” A qualitative study of the beliefs of women with chronic low back pain in relation to their painful experience. Howell (1994). A theoretical model for caring for women with chronic non‐malignant pain (CNP). Hwang et al. (2004). Lived experience of Korean women suffering from rheumatoid arthritis: a phenomenological approach. Ito and Pascual (2024). Exploring women's chronic disease experiences: A mixed‐methods analysis of endometriosis narratives. Johnson et al. (2024). “It Made Me Not Want to See him…”: The Role of Patient‐Provider Communication in Influencing Rural‐Dwelling Women Veterans' Motivation to Seek Health Care for Managing Chronic Pain. Juuso et al. (2011). Living with a double burden: Meanings of pain for women with fibromyalgia. Juuso et al. (2014). Meanings of Being Received and Met by Others as Experienced by Women With Fibromyalgia. Juuso et al. (2016). The Workplace Experiences of Women with Fibromyalgia. Kanter et al. (2017). Important role of physicians in addressing psychological aspects of interstitial cystitis/bladder pain syndrome (IC/BPS): A qualitative analysis. Kengen Traska et al. (2012). Strategies used for managing symptoms by women with fibromyalgia. Kirkham et al. (2015). Painting pain: An interpretative phenomenological analysis of representations of living with chronic pain. Knutsen et al. (2022). ‘The sofa is my base in daily life’: The experience of long‐term, pelvic girdle pain after giving birth. Lehti et al. (2017). Walking down ‘Via Dolorosa’ from primary health care to the specialty pain clinic—Patient and professional perceptions of inequity in rehabilitation of chronic pain. Lightbourne et al. (2024). Living With Endometriosis: A Reflexive Thematic Analysis Examining Women's Experiences With the Irish Health care Services. Löfgren et al. (2006). ‘A constant struggle’: Successful strategies of women in work despite fibromyalgia. Lo Monaco et al. (2024). The lived experience of mothers living with fibromyalgia syndrome: A phenomenological inquiry. Mellado et al. (2016). Social isolation in women with endometriosis and chronic pelvic pain. Mellado et al. (2020). Daily life attitudes of women with moderate or severe chronic pelvic pain. A qualitative study. Meriwether et al. (2022). Beliefs and narratives associated with the treatment of chronic pelvic pain in women.
Michaëlis et al. (2015). Quality of life and coping strategies among immigrant women living with pain in Denmark: A qualitative study. Molin et al. (2021). Grieving over the past and struggling forward—A qualitative study of women's experiences of chronic pain one year after childbirth. Molin et al. (2022). Disempowering women‐a mixed methods study exploring informational support about pain persisting after childbirth and its consequences. Molin et al. (2024). *The ignored pain* – experiences of encounters with health care from the perspective of women with pain persisting after childbirth: A qualitative study. Monsivais (2013). Decreasing the stigma burden of chronic pain. Müllersdorf et al. (2011). The magnitude of reciprocity in chronic pain management: Experiences of dispersed ethnic populations of Muslim women. Mustafa et al. (2020). The lived experiences of chronic pain among immigrant Indian‐Canadian women: A phenomenological analysis. Mustafa et al. (2024). Chronic pain experiences of immigrant Indian women in Canada: A photovoice exploration. Nortvedt et al. (2015). Caught in suffering bodies: A qualitative study of immigrant women on long‐term sick leave in Norway. Nortvedt et al. (2016). A lonely life‐A qualitative study of immigrant women on long‐term sick leave in Norway. Nyen and Tveit (2018). Symptoms without disease: Exploring experiences of non‐Western immigrant women living with chronic pain. Osborn and Smith (1998). The personal experience of chronic benign lower back pain: An interpretative phenomenological analysis. Park et al. (2022). ‘I worked until my body was broken’: an ethnomedical model of chronic pain among North Korean refugee women. Peppard et al. (2022). The Lived Experience of Military Women With Chronic Pain: A Phenomenological Study. Peterson et al. (2023). ‘It just stops me from living’: A qualitative study of losses experienced by women with self‐reported endometriosis. Pryma (2017). “Even my sister says I'm acting like a crazy to get a check”: Race, gender, and moral boundary‐work in women's claims of disabling chronic pain. Råheim and Håland (2006). Lived Experience of Chronic Pain and Fibromyalgia: Women's Stories From Daily Life. Reibel and Pearson (2017). Beyond the Pain: A Look into the Experiences of Women Living with Fibromyalgia Rice et al. (2024). Gendered worlds of pain: Women, marginalization, and chronic pain. Richardson (2005). Establishing the (extra)ordinary in chronic widespread pain. Roberto and Reynolds (2002). Older women's experiences with chronic pain: Daily challenges and self‐care practices Schaefer (1995). Struggling to maintain balance: a study of women living with fibromyalgia. Skuladottir and Halldorsdottir (2011). The quest for well‐being: self‐identified needs of women in chronic pain. Söderberg and Lundman (2001). Transitions experienced by women with fibromyalgia. Söderberg et al. (1999). Struggling for dignity: The meaning of women's experiences of living with fibromyalgia. Wade and Shantall (2003). The meaning of chronic pain: A phenomenological analysis. Werner and Malterud (2003). It is hard work behaving as a credible patient: Encounters between women with chronic pain and their doctors. Werner et al. (2004). ‘I am not the kind of woman who complains of everything’: illness stories on self and shame in women with chronic pain. Westergården et al. (2021). ‘Moving between living in the shadow of pain and living a life with the pain in the shadows’ – women's experiences of daily life with chronic widespread pain: A qualitative study. White and Seibold (2008). Walk a mile in my shoes: An auto‐ethnographic study. Wong et al. (2023). Challenges, Concerns, and Experiences of Community‐Dwelling Older Women with Chronic Low Back Pain‐A Qualitative Study in Hong Kong, China. Wuytack and Miller (2011). The lived experience of fibromyalgia in female patients, a phenomenological study. Zander et al. (2013). Struggling for sense of control: Everyday life with chronic pain for women of the Iraqi diaspora in Sweden.

### Quality appraisal

Quality of individual studies was assessed independently by the first author using the 14‐item National Institute of Clinical Excellence (NICE) quality appraisal checklist for qualitative studies (NICE, 2012; see Table 3), and a sample was independently evaluated by the fourth author. As there is no gold standard appraisal tool or any agreement on the value or approach to quality appraisal (Majid & Vanstone, 2018), this tool was selected as it is comprehensive and provides a guide to overall assessment. The questions are also designed in such a way that they can cover a wide range of qualitative research methods. According to the checklist, studies should be rated as “++” if all or most of the criteria have been met, “+” if some of the criteria have been met (but where it is unlikely to affect the conclusion) and “−” if few or no items on the checklist have been met.

**TABLE 3:** Quality appraisal using NICE guidance.

Study authors	NICE guidelines checklist numbers/overall assessment (OA) rating														
(Date)	1	2	3	4	5	6	7	8	9	10	11	12	13	14	0A
Ahlsen et al. (2014)	++	++	+	++	++	+	+	++	+	+	++	++	++	−	+
Allen et al. (2015)	++	++	+	++	+	+	++	+	+	++	+	++	++	++	+
Altun et al. (2023)	++	++	++	++	++	++	++	+	++	++	++	++	++	++	++
Arman et al. (2020)	++	++	++	++	+	++	++	++	++	++	++	++	++	++	++
Barnes et al. (2021)	++	++	++	++	++	++	++	+	+	++	++	++	+	++	++
Bostick et al. (2018)	++	++	+	+	+	+	++	++	++	++	++	++	++	++	++
Campbell et al. (2022)	++	++	++	++	+	+	++	+	+	+	++	++	+	++	+
Campeau (2018)	++	++	+	+	+	++	+	++	++	+	++	++	+	++	+
Caton et al. (2024)	++	++	++	++	+	++	++	+	++	++	++	++	++	++	++
Dickson and Kim (2003)	++	++	+	++	+	+	+	++	++	++	++	++	+	++	++
Driscoll et al. (2018)	++	++	++	++	+	+	++	++	++	++	++	++	++	++	++
Dysvik et al. (2013)	++	++	++	++	++	+	++	++	++	++	++	+	++	++	++
Evans and de Souza (2008)	++	++	+	+	−	++	+	+	+	+	+	++	+	++	+
Gonzalez et al. (2015)	++	++	+	++	++	+	+	++	+	+	++	++	++	++	++
Gullacksen and Lidbeck (2004)	++	++	+	++	+	+	+	+	++	+	++	++	++	++	+
Hallberg and Carlsson (1998)	++	++	+	++	+	+	+	++	++	+	++	++	+	++	+
Hallberg and Carlsson (2000)	++	++	++	++	+	+	+	++	++	+	++	++	++	+	++
Hervik et al. (2023)	++	++	++	++	+	+	++	+	++	++	++	++	++	++	++
Horment‐Lara et al. (2022)	++	++	++	++	+	++	++	++	++	++	++	++	++	++	++
Howell (1994)	++	++	++	+	+	++	++	++	++	++	++	++	+	+	++
Hwang et al. (2004)	++	++	+	++	+	+	+	++	++	++	++	++	++	+	++
Ito and Pascual (2024)	++	++	++	++	+	+	++	++	++	++	++	++	++	++	++
Johnson et al. (2024)	++	++	++	++	++	++	++	+	++	++	++	++	++	++	++
Juuso et al. (2011)	++	++	+	++	+	+	+	++	+	++	++	++	++	++	++
Juuso et al. (2014)	++	++	+	++	+	+	+	++	+	++	++	++	++	++	++
Juuso et al. (2016)	++	++	++	++	+	++	+	++	++	+	++	++	+	++	++
Kanter et al. (2017)	++	++	+	++	+	++	+	+	++	++	++	++	++	++	++
Kengen Traska et al. (2012)	++	++	+	++	+	+	++	+	++	++	++	++	++	++	++
Kirkham et al. (2015)	++	++	++	++	+	++	++	++	++	+	++	++	+	++	++
Knutsen et al. (2022)	++	++	+	++	++	+	++	++	++	++	++	++	+	++	++
Lehti et al. (2017)	++	++	++	++	++	+	++	+	++	++	++	++	++	++	++
Lightbourne et al. (2024)	++	++	++	++	++	+	++	+	++	++	++	++	++	++	++
Löfgren et al. (2006)	++	++	++	+	+	++	++	+	+	++	+	++	+	++	+
Lo Monaco et al. (2024)	++	++	++	++	++	+	++	++	++	++	++	++	++	++	++
Mellado et al. (2016)	++	++	++	++	+	++	++	++	++	++	++	++	++	++	++
Mellado et al. (2020)	++	++	+	++	+	+	+	++	++	++	++	++	++	++	++
Meriwether et al. (2022)	++	++	++	++	+	++	++	++	++	++	++	++	++	++	++
Michaëlis et al. (2015)	++	++	++	++	+	+	++	++	++	++	++	++	++	++	++
Molin et al. (2021)	++	++	++	++	+	+	++	++	++	++	++	++	+	++	++
Molin et al. (2022)	++	++	++	++	+	+	++	++	++	++	++	++	++	++	++
Molin et al. (2024)	++	++	++	++	+	+	++	+	++	++	++	++	++	++	++
Monsivais (2013)	++	++	+	+	+	+	++	+	+	+	++	++	+	+	+
Müllersdorf et al. (2011)	++	++	++	++	+	+	+	++	+	++	++	++	+	++	++
Mustafa et al. (2020)	++	++	++	++	+	++	+	+	++	++	++	++	++	++	++
Mustafa et al. (2024)	++	++	++	++	++	++	++	+	++	+	++	++	++	++	++
Nortvedt et al. (2015)	++	++	+	++	+	++	++	+	+	++	++	++	+	++	++
Nortvedt et al. (2016)	++	++	+	++	+	+	++	+	+	++	++	++	++	++	++
Nyen and Tveit (2018)	++	++	++	++	+	+	+	+	++	++	++	++	++	++	++
Osborn and Smith (1998)	++	++	+	++	+	+	+	++	++	++	++	++	+	−	+
Park et al. (2022)	++	++	++	++	++	+	++	+	++	+	++	++	++	++	++
Peppard et al. (2022)	++	++	+	++	+	+	++	++	++	+	++	++	++	++	++
Peterson et al. (2023)	++	++	++	++	++	+	++	++	++	++	++	++	++	++	++
Pryma (2017)	++	++	+	++	+	++	+	+	++	+	+	++	+	++	+
Råheim and Håland (2006)	++	++	++	++	+	++	+	++	++	++	++	++	+	++	++
Reibel and Pearson (2017)	++	++	++	++	++	+	++	+	++	++	++	++	++	++	++
Rice et al. (2024)	++	++	++	++	+	+	++	++	++	++	++	++	++	++	++
Richardson (2005)	++	++	++	++	+	+	+	−	++	+	++	++	+	++	+
Roberto and Reynolds (2002)	++	++	++	++	+	+	++	+	++	++	++	++	+	−	++
Schaefer (1995)	++	++	+	++	++	−	++	+	+	++	++	++	+	+	+
Skuladottir and Halldorsdottir (2011)	++	++	++	++	+	+	+	++	++	++	++	++	+	++	++
Söderberg and Lundman (2001)	++	++	+	++	+	+	+	+	++	++	++	++	+	++	+
Söderberg et al. (1999)	++	++	+	++	+	+	++	++	++	++	++	++	+	++	++
Wade and Shantall (2003)	++	++	+	++	+	+	+	+	++	+	++	++	+	+	+
Werner and Malterud (2003)	++	++	+	++	+	+	+	++	++	+	++	++	++	−	+
Werner et al. (2004)	++	++	+	++	+	+	+	+	+	+	+	+	+	−	+
Westergården et al. (2021)	++	++	+	++	+	+	+	++	++	++	++	++	++	++	++
White and Seibold (2008)	++	++	++	++	+	+	+	+	+	+	++	++	+	++	+
Wong et al. (2023)	++	++	++	++	+	+	++	+	++	++	++	++	++	++	++
Wuytack and Miller (2011)	++	++	+	++	+	+	+	++	++	+	++	++	++	++	++
Zander et al. (2013)	++	++	+	++	+	++	++	++	+	++	+	++	++	++	++

*Note*: Appraisal Key: (++ = Appropriate/Sufficient, − = Insufficient, + = Not Sure/Mixed). 1. Is a qualitative approach appropriate? (Appropriate (++), Inappropriate (−), Not sure (+)). 2. Is the study clear in what it seeks to do? (Clear (++), Unclear (−), Mixed (+)). 3. How defensible/rigorous is the research design/methodology? (Defensible (++), Indefensible (−), Not sure (+)). 4. How well was the data collection carried out? (Appropriately (++), Inappropriately (−), Not sure/inadequately reported (+)). 5. Is the role of the researcher clearly described? (Clearly described (++), Unclear (+), Not described (−)). 6. Is the context clearly described? (Clear (++), Unclear (−), Not sure (+)). 7. Were the methods reliable? (Reliable (++), Unreliable (−), Not sure (+)). 8. Is the data analysis sufficiently rigorous? (Rigorous (++), Not rigorous (−), Not sure/not reported (+)). 9. Is the data ‘rich’? (Rich (++), Poor (−), Not sure/not reported (+)). 10. Is the analysis reliable? (Reliable (++), Unreliable (−), Not sure/not reported (+)). 11. Are the findings convincing? (Convincing (++), Not convincing (−), Not sure (+)). 12. Are the findings relevant to the aims of the study? (Relevant (++), Irrelevant (−), Partially relevant (+)). 13. Is there adequate discussion of any limitations encountered? (Adequate (++), Inadequate (−), Not sure (+)). 14. How clear and coherent is the reporting of ethics? (Clear (++), Unclear (−), Not sure (+)). Overall assessment (OA): As far as can be ascertained from the paper, how well was the study conducted? ((++) All or most of the checklist criteria have been fulfilled, where they have not been fulfilled the conclusions are very unlikely to alter. (+) Some of the checklist criteria have been fulfilled, where they have not been fulfilled, or not adequately described, the conclusions are unlikely to alter. (–) Few or no checklist criteria have been fulfilled and the conclusions are likely or very likely to alter).

Validity and confidence in the review findings were assessed using the GRADE‐CERQual approach (Grading of Recommendations Assessment, Development and Evaluation—Confidence in the Evidence from Reviews of Qualitative research; Lewin et al., 2018). The GRADE‐CERQual approach comprises four components: methodological limitations, coherence (an assessment of the fit between primary study data and the review findings: consistency across studies), adequacy of data (a measure of how rich and well supported a review finding is by the included studies) and relevance (how well the evidence from the primary studies fits into the context of the review question; Lewin et al., 2018).

The first dimension, methodological limitations, was assessed using the NICE checklist based on the quality ratings of the studies included in the review. Relevance was assessed according to how well the studies addressed the review question (inclusion criteria), which specified the experience of females over the age of eighteen living with chronic pain in any setting. Coherence involved assessing how clearly the review findings represented a strong fit with the underlying data from the primary studies. During the analysis stage, when themes were selected and synthesized, the consistency of the data and the extent to which the data clearly supported the review findings were examined. Adequacy examined the richness of the data and the number of studies supporting a review finding.

### Data extraction and synthesis

A standardized data extraction sheet was used to extract the following: research title, author, date, country, aims, sample size, participant details (ethnicity, condition, age range of women), data collection method, data analysis method and summary of key findings/themes (presented in Table 4). All data from the included studies under the headings abstract, results or findings, discussion and conclusion were regarded as data.

**TABLE 4:** Overview of the studies included in the review.

Study title, author, date and (country)	GRADE‐CERQual ID	Aims of the study	Details of female participants (ethnicity, condition, age range of women)sample size (women)	Data collection method/analysis method	Key findings/themes
Ahlsen et al. (2014). (Un)doing gender in a rehabilitation context: A narrative analysis of gender and self in stories of chronic muscle pain (Norway)	1	Exploration of gender in the self‐told stories of men and women undergoing rehabilitation for chronic pain	6 Norwegian Women with Chronic Neck Pain 28–50 years	Interviews Narrative Analysis	The evolving self through rehabilitation: women's stories tended to develop from “chaos”, towards a quest narrative with a more autonomous self. Their stories displayed selves that were actively trying to transcend their former identity and life conditions, in which their pain was embedded
2 Allen et al. (2015). Exploring the experience of chronic pain among female Survival Sex Workers: a qualitative study (Canada)	2	To understand the experience of chronic pain among female Survival Sex Workers (and address the gap in literature on chronic pain experience in this group) in Vancouver's downtown east side	11 females with chronic pain Age 42–56 years Six women identified as Aboriginal and five as Caucasian	In‐depth semi‐structured interviews Thematic Analysis	1. Communication understanding chronic pain (subjectivity and diversity in descriptions) communicating with others: not being heard 2. Cures Use of various substances to numb the pain 3. Systemic barriers multiple systemic barriers were described by women in managing their chronic pain (including judgement (stigma) and poverty). 4. Stressors (adverse experiences in life/lack of family support). 5. Support: peer support groups were vital
3 Altun et al. (2023). Experiences of Assyrian refugee women seeking care for chronic pain: a qualitative study. *International Journal for Equity in Health*, *22*(1), 83 (Australia)	57	The study aimed to explore the lived experiences of Assyrian refugee women seeking care for chronic pain, focusing on their health‐seeking narratives and the factors influencing their access to care	The sample consisted of 10 Iraqi refugee women aged between 19 and 85 years, all of whom had lived in Australia for varying durations (between 4 and 25 years). They had experienced chronic pain for over three months	Semi‐structured, in‐depth interviews Phenomenological approach	(1) The story of pain; (2) the experience of helpseeking in Australia and the home country; (3) factors shaping the ability to access appropriate care; (4) support seeking systems; and (5) influence of culture and gender roles
4 Arman et al. (2020). Women's Lived Experiences of Chronic Pain: Faces of Gendered Suffering (Sweden)	3	To understand the lived experience of women with chronic pain (from a caring science and gender perspective)	21 women with Chronic pain 20–61 years	Semi‐structured interviews Hermeneutic Phenomenology (Gadamerian Hermeneutics)	1. Living an Overwhelming Life in Loneliness 2. Taking Care of Others Without Yourself Being Cared For 3. Keep Going and a “Knackered” Body 4. Understanding Suffering in the Light of Their Own Lives
5 Barnes et al. (2021). Exploring the emotional experiences of young women with chronic pain: The potential role of self‐compassion (Canada)	4	Exploration of the emotional experiences of women with chronic pain and the role of self‐compassion	Seven women with chronic pain that was not related to another heath condition or related to a diagnosed pain condition Age 19–34 years All Canadian and white (one also self‐identified as indigenous)	Narrative Inquiry Focus groups Reflective photo voice One‐to‐one semi‐ structured interviews Holistic‐content analysis	1. The Emotional Challenges of Chronic Pain 2. The Journey to Self‐Awareness 3. The Transition to a Self‐Compassionate Mindset
6 Bostick et al. (2018). Pain Assessment Recommendations for Women, Made by Women: A Mixed Methods Study (Canada)	5	To Qualitatively explain unique features of women's pain experiences	10 women living with chronic (non‐cancer) pain 24–63 years	Semi‐structured (phone) interviews (qualitative phase) Content Analysis	Qualitative findings 1. Stigmatization (not being listened to; Pain is more than a number; and social roles & norms)
7 Campbell et al. (2022). Women's experiences of navigating chronic pain within the context of living with an episodic disability (Canada)	6	To explore the experience of chronic pain for women living with episodic disabilities	30 women Age 20–62 years living with chronic pain and an episodic disability (Lupus, Multiple Sclerosis, Fibromyalgia, Ehlers‐Danlos syndrome, Rheumatoid arthritis and irritable bowel syndrome)	Semi‐structured interviews Interpretive description	Navigating and responding to the health care system The power of being dismissed Dis(respecting) medical advice Seeking personalized care in an unpersonal system. The “cost” of well‐being. Seeking the essentials of living beyond health care
8 Campeau (2018). Adaptive frameworks of chronic pain: Daily remakings of pain and care at a Somali refugee women's health centre (United States)	7	To understand Somali women's use of informal and formal networks of health care	12 Somali women living with chronic pain (headaches *n* = 5, Lupus *n* = 2, auto‐immune disease *n* = 2, post‐traumatic stress disorder & Endometriosis *n* = 1, Chronic pelvic and back pain *n* = 1 and Rheumatoid arthritis and heart disease, *n* = 1) Interviews (*n* = 12) and Focus groups (*n* = 8), participant observation (12 women with pain and 3 health educators) Age 29–56 years	Ethnographic study: Interviews, Focus groups and Participant observations Grounded Theory	Four frameworks identified: 1. Pain as a symptom of exile (lost home linked to bygone health) 2. Pain and the strength to bear pain as issues of faith (faith as a coping strategy for all the participants) 3. Medicine as powerful, curative and fluid 4. Medical discrimination and exclusion
9 Caton et al. (2024). “My Goal is…to get Through the Day Without Pain”: A Qualitative Study on Chronic Pain Experiences and Treatment Needs Among Child Caregiving Women (United States)	58	To understand connections between pain, caregiving, physical and behavioural health treatment needs and motivations for prescription opioid use among child caregiving women with chronic pain	12 women with CP (Age: 35–66 years; 8 Black; 3 White and 1 Asian) in child caregiving roles, including women who were pregnant or trying to become pregnant and were caring for children or grandchildren <18 years, receiving treatment at an outpatient pain management clinic	Semi structured interviews Thematic analysis	Key Findings: Experiences of pain and trauma; the impact of pain on emotional and mental health, daily life and relationships; health care experiences; the use of prescription opioids; and sources of support
10 Dickson and Kim (2003). Reconstructing a meaning of pain: older Korean American women's experiences with the pain of osteoarthritis (United States)	8	To gain a deeper understanding of older Korean American women's experiences of chronic osteoarthritic pain	Women (*n* = 7) with Osteoarthritis Age 63–80 years Korean American	Interviews Grounded Theory	Women came to perceive their pain as a component off ageing rather than as a symptom of disease Five stage process of constructing meaning of pain: Suffering with pain Struggling to remove pain Stumbling along with pain Striving to reduce pain Managing and tolerating pain
11 Driscoll et al. (2018). Patient Experiences Navigating Chronic Pain Management in an Integrated Health Care System: A Qualitative Investigation of Women and Men (United States)	9	To describe perceptions of managing pain in an integrated health care system and to explore gender differences	Women (*n* = 22) with chronic pain Average age 55.2 years 64% White	Focus groups Grounded Theory	1. Just keep plugging a. always a. reacquaintance process b. so many hoops c. to medicate or not. A distinct theme, “the challenges of being female,” reflected women's perceptions of stigma and bias
12 Dysvik et al. (2013). A narrative approach to explore grief experiences and treatment adherence in people with chronic pain after participation in a pain‐management program: A 6‐year follow‐up study (Norway)	10	To explore grief caused by chronic pain and treatment adherence and how these experiences are integrated into ongoing life stories	Five women living with chronic pain caused by different musculoskeletal disorders Age 41–66 years	Narrative inquiry (image/written narratives) Narrative Analysis	Experiences of grief over time were commonly associated with chronic pain. The participants' past experiences reflected their grief at having to abandon jobs and social networks and revealed loneliness and despair. Adaptation and hope for the future had been established
13 Evans and de Souza (2008). Dealing with chronic pain: Giving voice to the experiences of mothers with chronic pain and their children (New Zealand)	11	Exploration of the impact of chronic pain on mothers and their children (from a gains and losses theory and the strengths perspective)	16 mothers with chronic pain Age 27–45 years (Lupus *n* = 1, arthritis *n* = 1, polycystic ovary syndrome *n* = 1, migraine *n* = 2, repetitive strain injury *n* = 2, spinal pain *n* = 9) All White sample	Open‐ended semi‐structured interviews Framework (deductive) Analysis	Pain formed a substantial part of the participants' lives. Every facet of life was impacted by pain. Positive and negative aspects of pain were identified (pain as a burden and focus on blessings). Parenting was observed as a two‐way interaction
14 Gonzalez et al. (2015). Life History of Women with Fibromyalgia: Beyond the Illness (Portugal)	12	To explore the life history of women with fibromyalgia that had experienced a critical or very stressful life event before the onset of the syndrome	10 women with fibromyalgia Ages 29–59 years	Interviews Interpretative Phenomenological Analysis	1. Struggle, 2. Focus on adversities, 3. Positive overlaps the negative, 4. Scars of an unhappy childhood, 5. Help others, 6. Perfectionism and desire to achieve, 7. unsatisfactory present, 8. perception of injustice and 9. keep feelings inside
15 Gullacksen and Lidbeck (2004). The life adjustment process in chronic pain: psychosocial assessment and clinical implications (Sweden)	13	To explore the subjective experience/life adjustment processes of women diagnosed with chronic musculoskeletal pain	18 women (11 with myofascial pain syndrome and 7 with fibromyalgia) Age 23–55 years	Interviews Inductive thematic analysis (phenomenological framework)	A proposed model for life adjustment Three stages of the life adjustment process: 1. Prelude, struggling to restore life, self‐deception, confirmation and acknowledgement 2. Working through, sorrow and loss, losing oneself, leaving the role of being sick, defining the problems, finding solutions, picture of the future affects coping. 3. Establishing a new course of life. After the three stages, the work of maintaining the adjustment reached is continuous
16 Hallberg and Carlsson (1998). Psychosocial vulnerability and maintaining forces related to fibromyalgia. In‐depth interviews with twenty‐two female patients (Sweden)	14	To describe women's experiences of living with Fibromyalgia, their beliefs about the pain and its origin and how pain impacts family and social life To broaden the understanding of what it means to the women to be living with Fibromyalgia	22 women with Fibromyalgia Age 22–60 years	Open‐ended in‐depth interviews Grounded Theory	Main findings Psychosocial vulnerability (with subcategories: traumatic life history, over‐compensatory perseverance, pessimistic life view and unsatisfying work situation, which contribute to the development of CP) and maintaining forces (three subcategories: professional care, pain benefits and family support, which contribute to the persistence and chronicity of the pain symptoms)
17 Hallberg and Carlsson (2000). Coping with fibromyalgia: A qualitative study (Sweden)	15	To describe women's experiences of living with chronic pain (diagnosed with fibromyalgia) and how they manage their situation	22 women with Fibromyalgia Age 22–60 years	Open‐ended in‐depth interviews Grounded theory	Core concept: preoccupied with pain Three categories: subjective pain language diversified pain coping pain communication
18 Hervik et al. (2023). Living with chronic headaches: A qualitative study from an outpatient pain clinic in Norway (Norway)	59	The study aimed to explore the lived experiences of individuals suffering from chronic headaches	12 women with chronic headaches (38–68 years)	Semi‐structured interviews Thematic Analysis	The following domains were identified: emotions related to headaches; trauma/stressful events; behavioural changes; relationships and coping mechanisms. The complexity of this pain in clinical practice is hugely underestimated and poorly understood by society in general
19 Horment‐Lara et al. (2022). “I don't want to be a burden” A qualitative study of the beliefs of women with chronic low back pain in relation to their painful experience (Chile)	16	To explore the beliefs of women with non‐specific chronic low back pain (regarding the nature of their symptoms, fears associated with pain, expectations for recovery, family, social and work‐related limitations and perceived self‐efficacy)	10 women with non‐specific chronic low back pain Age 43–76 years	Semi‐structured interviews Thematic (deductive) analysis	1. Beliefs regarding the nature of pain 2. Fears associated with the experience of pain 3. Expectations of recovery 4. Behavioural outcomes originating from beliefs: social life and self‐efficacy
20 Howell (1994). A theoretical model for caring for women with chronic non‐malignant pain (CNP) (United States)	17	To uncover the underlying processes in the experience of living with CNP from women's perspectives and explain these processes	19 women living with chronic pain (low back pain, phantom limb pain, fibromyalgia, arthritis and migraine headaches) Age 21–76 years (Details on ethnicity provided for fourteen participants: 11 Caucasian, 2 Hispanic and 1 Black)	In‐depth Interviews Participant observation Critical incident health diaries Grounded Theory	Theory to explain women's healthy progression through the experience of living with CNP (three phases): 1. The pain takes over 2. Filling my life with new hope (some women did not progress to this healthy phase but progressed to illness: ‘filling my life with pain and despair’). Progression is influenced by patterns of validating (others/doctors). 3. Fulfilling my life with pain
21 Hwang et al. (2004). Lived experience of Korean women suffering from rheumatoid arthritis: a phenomenological approach (Korea)	18	To explore and describe the illness experience of women with Rheumatoid arthritis in Korea	5 women with Rheumatoid arthritis 34–61 years	Interviews Phenomenology	1. severe pain 2. self‐esteem 3. negative feelings 4. reflect the past life 5. concentrate on recovery from disease 6. a comfortable mind in pain 7. support of family and others 8. new life
22 Ito and Pascual (2024). Exploring women's chronic disease experiences: A mixed‐methods analysis of endometriosis narratives (Chile)	60	The study aims to fill the gap in understanding the endometriosis experience of Latin American women	30 Women (23–47 years) Chilean women with endometriosis	Semi‐open interviews Content Analysis Natural language processing technique to extract and analyse participants' narratives	By focusing on the keywords “pain,” “endometriosis,” and “menstruation,” the NLP analysis identified nine semantic domains related to the pain experience Nine semantic domains of the endometriosis pain experience: intensity, normalization, treatment, frequency, menstruation, feeling, pain location, symptoms and impact
23 Johnson et al. (2024). “It Made Me Not Want to See him…”: The Role of Patient‐Provider Communication in Influencing Rural‐Dwelling Women Veterans' Motivation to Seek Health Care for Managing Chronic Pain (United States)		The study aimed to understand the priorities and experiences of rural‐dwelling women Veterans in using health care for managing their chronic pain. Specifically, it sought to explore the factors influencing their decision‐making and motivations for seeking care	16 participants, ranging from 35 to 63 years old, experienced chronic pain for an average of 21 years Fourteen participants identified as White, one participant each identified as Black and American Indian	Interviews Thematic Analysis/Grounded Approach informed by Self‐Determination Theory	Motivations for seeking chronic pain care: Competing Priorities: Women described balancing their health care needs with other responsibilities such as work, education and family care. However, most expressed a desire to function effectively in their daily life and relationships Role of Trust: Trust in the provider was identified as a key motivational factor Feeling dismissed by HCPs and the role of gender in feeling dismissed. Women also discussed distrusting their providers. For many participants, feeling dismissed by their providers was understood as an issue related to gender
24 Juuso et al. (2011). Living with a double burden: Meanings of pain for women with fibromyalgia (Sweden)	19	To elucidate the meanings of pain for women with Fibromyalgia	15 women with Fibromyalgia Age 38–64 years	Interviews Phenomenology	1. Experiencing an unwilling body and 2. Experiencing a good life despite all Pain dominated daily life, but the women found ways to manage and control it. They experienced disbelief/not being taken seriously and found relief through distraction
25 Juuso et al. (2014). Meanings of Being Received and Met by Others as Experienced by Women with Fibromyalgia (Sweden)	20	To elucidate meanings of being received and met by others as experienced by women with FM	9 women with fibromyalgia Age 40–65 years	Interviews Phenomenology	1. Being seen as a malingerer 2. Being acknowledged Meanings of being received and met by others, as experienced by women with FM, was seen as a movement between the two perspectives. When women were acknowledged, their feelings of security and trust increased. However, the women could not rely on this because others received and met them in such an unpredictable manner
26 Juuso et al. (2016). The Workplace Experiences of Women with Fibromyalgia (Sweden)	21	To explore experiences of the workplace of women living with Fibromyalgia	15 women Age 38–64 years	In‐depth interviews Hermeneutic Phenomenology (Gadamerian Hermeneutics)	1. The body as an obstacle to working (women wished to work but found it exhausting) 2. Accepting the inability to work as before 3. Work meant everything in life 4. A future shaped in vagueness
27 Kanter et al. (2017). Important role of physicians in addressing psychological aspects of interstitial cystitis/bladder pain syndrome (IC/BPS): A qualitative analysis (United States)	22	To explore patients' experience with Interstitial cystitis/bladder pain syndrome symptoms and of their medical care to elicit suggestions to improve patient satisfaction with that care	15 women with Interstitial cystitis/bladder pain syndrome (Mean age 52.6 years) American Indian/Alaskan Native (*n* = 2), Non‐Hispanic Caucasian (*n* = 6), Hispanic (*n* = 4), Other (*n* = 3)	Focus groups Grounded Theory	1. IC/BPS is a life‐altering, debilitating condition 2. Fear and anxiety from the unrelenting and unpredictable nature of the disease 3. Isolation Provider impact 1. Patients wanted to know that all their providers (irrespective of whether they were physicians, nurses, physical therapists, or otherwise) were truly listening to them. 2. Participants desired increased knowledge about their condition and largely preferred to hear about treatment options. 3. Provider expression of hope for improvement of their symptoms was vital
28 Kengen Traska et al. (2012). Strategies used for managing symptoms by women with fibromyalgia (United States)	23	To describe how individuals with fibromyalgia manage their condition and explore the strategies they utilize	8 women with fibromyalgia Age 54–81 years Hispanic, *n* = 1 and Caucasian, *n* = 7	Focus group Content Analysis	(1) pacing/planning; (2) focusing on mind, body and spirit/distraction; (3) coping with sensitivity to touch; (4) social support; (5) pushing yourself/putting on a mask; and (6) medications
29 Kirkham et al. (2015). Painting pain: An interpretative phenomenological analysis of representations of living with chronic pain (England)	24	To understand women's experiences of living with chronic pain and their conditions through pictorial representations of their chronic pain, alongside their accounts of those images	Seven women living with chronic pain White British Age 36–52 years	Semi‐structured Interviews/illustrations Interpretative Phenomenological Analysis	1. Pain as an object: Sinister, violent, punitive 2. The colour of pain: Red and Burning, Black and Brooding Images showed a movement from the self before pain to the self since the pain had started or pointing to aspirations for the possible relief of pain in the future
30 Knutsen et al. (2022). ‘The sofa is my base in daily life’: The experience of long‐term, pelvic girdle pain after giving birth (Norway)	25	To explore how women struggling with long‐term pelvic girdle pain after giving birth experienced it and coped in their daily life	9 Norwegian women with pelvic girdle pain Age 26–56 years	Semi‐structured interviews Phenomenology	1. A life with pain and an unpredictable body 2. An identity as disabled, dependent and ashamed 3. Recharging on the sofa: adaptation and fighting for dignity and acceptance 4. Striving to live as you wish: isolation and working life
31 Lehti et al. (2017). Walking down ‘Via Dolorosa’ from primary health care to the specialty pain clinic—Patient and professional perceptions of inequity in rehabilitation of chronic pain (Sweden)	26	To analyse patient and professional perceptions about (in)equity of care and rehabilitation of chronic pain patients from primary health care to assessment at a specialty rehabilitation clinic	(sample of women) 5 females living with chronic pain Age: 35–65 years	Focus group and semi‐structured interviews Grounded Theory	one core category, ‘walking down Via Dolorosa’ and five inter‐related categories ‘pain – an illness with low status’, ‘stereotyping thoughts’, ‘burdened by pain – referrals as a way out’, ‘assessing and selecting in context’ and ‘a proper patient, ready to change’
32 Lightbourne et al. (2024). Living With Endometriosis: A Reflexive Thematic Analysis Examining Women's Experiences With the Irish Healthcare Services (Ireland)	62	To explore the perceptions and experiences of women with endometriosis regarding the diagnosis, support and treatment options available in Ireland	20 women with endometriosis and having experience of the health care services in Ireland Age 18 to 50s (late)	Online semi‐structured interviews Reflexive thematic analysis	Key Findings: (1) Dismissive attitudes normalizing severe pain (2) Inadequate health system (3) The impact of delayed diagnoses (4) Lack of education and awareness (5) Navigating ignorance, taboo and societal views
33 Löfgren et al. (2006). ‘A constant struggle’: Successful strategies of women in work despite fibromyalgia (Sweden)	27	To explore and obtain increased knowledge of the strategies used by working women with fibromyalgia regarding control of pain, fatigue and other symptoms.	12 females living with fibromyalgia Age 30–63 years	Diaries and focus groups (*n* = 7) and individual interviews(*n* = 2), diary (*n* = 3) only and FG only (*n* = 2) Content analysis and Grounded Theory	The core category ‘constant struggle’ with eight subcategories: enjoying life, taking care of oneself, positive thinking, setting limits, using pain as a guide, creative solutions, learning/being knowledgeable and ‘walking a tightrope’. A ‘grieving process’ was a prerequisite for managing the struggle and ‘social support’ facilitated the struggle
34 Lo Monaco et al. (2024). The lived experience of mothers living with fibromyalgia syndrome: A phenomenological inquiry (Italy)	63	To explore the lived experiences of motherhood and daily life among women diagnosed with fibromyalgia	10 Italian mothers with fibromyalgia Age 34–60 years	Semi‐structured interviews Phenomenological study using Colaizzi's method	Five themes: A trauma preceding diagnosis, Pervasive feelings of misunderstanding, A struggle to maintain strength among limitations, Challenges in fulfilling maternal roles and Persistent sexual discomfort
35 Mellado et al. (2016). Social isolation in women with endometriosis and chronic pelvic pain (Brazil)	28	To evaluate the perceptions of women with endometriosis and chronic pelvic pain regarding their social ties	29 females with endometriosis and chronic pelvic pain Age 21–49 years	Focus groups Grounded Theory	1. Social isolation a. Avoiding intimacy b. isolation from family and friends c. Lack of understanding about the disease d. Resignation
36 Mellado et al. (2020). Daily life attitudes of women with moderate or severe chronic pelvic pain. A qualitative study (Brazil)	29	To understand the attitudes adopted by women with chronic pelvic pain (CPP) to deal with daily life problems caused by their condition	58 females with CPP Age 22–57 years	Semi‐structured interviews (phenomenological study) Thematic Analysis	(1) shaping life by pain; (2) isolating from social contact; (3) avoiding sexual relationships; (4) seeking pain relief; (5) seeking positive strategies (these were more frequent in older women)
37 Meriwether et al. (2022). Beliefs and narratives associated with the treatment of chronic pelvic pain in women (United States)	64	To obtain diverse stakeholder perspectives on CPP treatment	48 patients with CPP (19 in discussion groups, 29 in individual interviews; age 40 ± 12) Most participants identified as Hispanic/Latina or non‐Hispanic White. Hispanic/Latina (25) White (12), Native American (2), Asian (1) Black (2) Native Hawaiian/Pacific Islander (1). The remaining in the unknown/not reported category	Interviews/discussion groups Thematic Analysis/grounded theory methodology	Five Key themes: Physical debilitation of pain Emotional toll of pain Challenges in health care interactions Options and considerations for CPP treatment The value of not feeling alone
38 Michaëlis et al. (2015). Quality of life and coping strategies among immigrant women living with pain in Denmark: A qualitative study (Denmark)	30	To examine the quality of life and coping strategies among immigrant women living with chronic pain in Denmark	13 females living with chronic pain. All participants were non‐western immigrant women who had migrated to Denmark from Turkey, Iraq, Afghanistan, Somalia, Pakistan, Jordan or Morocco Age 33–63 years	Semi‐structured interviews (and observations) Content Analysis	1. Experiences of chronic pain (the negative impact on activities of daily living, altered mental well‐being, strained social relations) 2. Coping with chronic pain (altering everyday life, seeking health care)
39 Molin et al. (2021). Grieving over the past and struggling forward—A qualitative study of women's experiences of chronic pain one year after childbirth (Sweden)	31	To describe women's experiences of chronic pain related to childbirth approximately one year after labour	20 women experiencing chronic pain Age 25–43 years	In‐depth interviews Content analysis	1.“Grieving over the past and struggling forward” a. “Mourning the losses”, b. “Struggling with the present” and c. “Managing the future”
40 Molin et al. (2022). Disempowering women‐a mixed methods study exploring informational support about pain persisting after childbirth and its consequences (Sweden)	32	To explore women's experience and thoughts regarding information about chronic pain and informational support about pain persisting after childbirth and its consequences	20 women experiencing chronic pain Age 25–43 years (in‐depth sample)	In‐depth interviews (qualitative phase) Content analysis	Three categories emerged in the interview data: 1. “Inadequate information”, 2. “Negative consequences (emotional distress)” and 3. “Information needs and requirements”
41 Molin et al. (2024). *The ignored pain* – experiences of encounters with healthcare from the perspective of women with pain persisting after childbirth: A qualitative study (Sweden)	65	The study aimed to explore how women with pain persisting for at least eight months after childbirth experienced encounters with health care	20 women (25–43 years) living with chronic pain (persisting after childbirth)	Semi‐structured interviews Inductive qualitative content analysis	“Pain ignored by healthcare” was identified as a central theme from the interviews. This included four categories: “Questioned pain experience,” “Inadequate pain management,” “Lost in healthcare,” and “Insufficient postpartum care”
42 Monsivais (2013). Decreasing the stigma burden of chronic pain (United States)	33	To explore stigmatizing experiences shared by Mexican American women living with chronic pain and provide guidelines for reducing stigma	15 Mexican American women 21–65 years old living with chronic pain	Ethnographic study consisting of semi structured interviews, participant observations and fieldwork Thematic analysis	1. Role functions and communication within the family (participants were often unable to fulfil role functions within the family because of pain) 2. Role functions and communication within the workplace (women remained silent about the pain in order to maintain their identities as independent, truthful and hardworking women). To reduce stigma HCPs must understand their own misconceptions about CP
43 Müllersdorf et al. (2011). The magnitude of reciprocity in chronic pain management: Experiences of dispersed ethnic populations of Muslim women (Sweden)	34	To examine the experience of living with musculoskeletal pain and experience of health care among dispersed ethnic populations of Muslim women	Five Females with chronic pain (MSK) Age 33–56 years	Semi‐structured interviews Grounded Theory	1. ‘The magnitude of reciprocity’ based on a. impact of pain, b. managing pain and c. facing health care
44 Mustafa et al. (2020). The lived experiences of chronic pain among immigrant Indian‐Canadian women: A phenomenological analysis (Canada)	35	To explore the lived experiences of chronic pain among immigrant Indian women in Canada	Thirteen women with chronic musculoskeletal pain Age 34–60 years Indian	Semi‐structured interviews Thematic analysis (informed by van Manen's phenomenology of practice)	1. The body in pain 2. Pain in the context of lived and felt space 3. Pain and Relationships 4. Pain and time
45 Mustafa et al. (2024). Chronic pain experiences of immigrant Indian women in Canada: A photovoice exploration (Canada)	66	To explore immigrant Indian women's chronic pain experiences in Canada and aimed to enhance the understanding of those experiences by creating a visual opportunity for them to share their stories	Twelve immigrant Indian women living with chronic pain (MSK). Age: 34–60 years	Photovoice/discussion Phenomenological reflection/thematic analysis	(1) bodies in pain, (2) traversing spaces including immigration and (3) pain management methods
46 Nortvedt et al. (2015). Caught in suffering bodies: A qualitative study of immigrant women on long‐term sick leave in Norway (Norway)	36	To explore the issues faced by immigrant women on long‐term sick leave due to chronic pain, focusing on their personal perspectives on their daily lives, their bodies and their pain	14 females (immigrants from Asia/Africa) with chronic pain Age 30–59 years	Participant observation (*n* = 14) and in‐depth interviews (*n* = 11) Phenomenology	1. Bodies marked by onerous experience (two subthemes: It is in my body and Invisible pain)
47 Nortvedt et al. (2016). A lonely life‐A qualitative study of immigrant women on long‐term sick leave in Norway (Norway)	37	To explore how immigrant women on long‐term sick leave in Norway due to chronic pain experience their illness and their relationships at work and in the family	14 females (immigrants from Asia/Africa) with chronic pain Age 30–56 years	Participant observation (*n* = 14) and semi‐structured Interviews (*n* = 11) Phenomenology	1. A lonely life Shut inside the home and rejected at the workplace
48 Nyen and Tveit (2018). Symptoms without disease: Exploring experiences of non‐Western immigrant women living with chronic pain (Norway)	38	The aims of the researchers were to explore the experiences of non‐Western immigrant women living with chronic pain in Norway	9 females (immigrant women from Pakistan, Morocco, Tunisia, Iran, Somalia and Ethiopia) with chronic pain Age 31–55 years	Semi‐structured interviews Phenomenology	1. Explaining pain 2. stressful lives 3. Painful Losses
49 Osborn and Smith (1998). The personal experience of chronic benign lower back pain: An interpretative phenomenological analysis (England)	39	To gain insight into the personal experience of chronic benign lower back pain	9 women with chronic back pain Age 25–55 years	semi‐structured interviews Interpretative Phenomenological Analysis	1. Searching for an explanation 2. Comparing this self with other selves 3. Not being believed and 4. withdrawing from others
50 Park et al. (2022). ‘I worked until my body was broken’: an ethnomedical model of chronic pain among North Korean refugee women Korea (South)	67	To explore the lived experiences of chronic pain among North Korean refugee (NKR) women in South Korea	20 NKR women, mean age 50.3 years (range 19–74), chronic pain, 11 participants reported musculoskeletal pain including back pain, shoulder pain and hip pain. Five women had headache and four had dyspepsia	Semi‐structured individual interviews Thematic Analysis	Key findings: Characteristics of chronic pain experienced Physical Factors as primary Causes of Pain (labour fatigue, malnutrition, exposure to violence) Psychological factors as collateral causes of pain Participants commonly ruminated on traumatic past experiences (such as loss and violence), which often triggered headaches or increased pain. Loneliness and stress related to family in North Korea were also noted as psychological contributors to physical pain. Pain management experiences and care expectations: Participants often felt that providers' attitudes were unsympathetic, leading to dissatisfaction with medical care and feelings of discrimination
51 Peppard et al. (2022). The Lived Experience of Military Women with Chronic Pain: A Phenomenological Study (United States)	40	To explore a typical day for military women living with chronic pain by examining the participants' daily life experiences	Thirteen active duty, retired, or veteran women experiencing chronic pain. Age 31–65 years Ethnicity Black or African‐American (*n* = 4), Hispanic or Latino (*n* = 1), Asian‐American/Pacific Islander(*n* = 1), White or Caucasian (*n* = 7)	Semi‐structured interviews Phenomenology	(1) living with chronic pain is frustrating, persistent, daily and an hourly struggle; (2) resilience in living with chronic pain is the new normal; (3) mission first and the impact of invisible pain;(4) self‐care management and the internal locus of control in using non‐pharmacological therapies; (5) pain accepted and managed to improve quality of life; (6) coronavirus disease(COVID‐19) diminished social interactions; (7) pain of sexual trauma is not reported; and (8) disparities in health care due to self‐perception of provider bias as pain is not understood
52 Peterson et al. (2023). ‘It just stops me from living’: A qualitative study of losses experienced by women with self‐reported endometriosis (Australia)	68	To understand the experience of loss in Australian women with endometriosis	532 Australian women aged between 18 and 50 years (M = 30.8, SD = 7.1) with a self‐ reported diagnosis of endometriosis	Online surveys Inductive Template Analysis	Three main themes were identified: the loss of liberty: ‘I'm trapped in the house’; the loss of bodily autonomy: ‘I can barely move/breathe/talk’ and loss of connection: ‘It stops me from being social’. Pain emerged as the greatest concern for participants, preventing them from the physical functioning required to participate in many of life's activities. Losses were often unacknowledged by loved ones and health care providers, further impacting the physical, emotional and mental health of participants
53 Pryma (2017). “Even my sister says I'm acting like a crazy to get a check”: Race, gender, and moral boundary‐work in women's claims of disabling chronic pain (United States)	41	To explore how the experiences of women with fibromyalgia who seek legitimation for their pain and disability change based on race. Also, how race shapes the moral boundary‐work performed by women suffering from disabling chronic pain	24 females with fibromyalgia Age late‐twenties to mid‐Sixties stated Ten identified as Black and/or African‐American, three as Latina, one as Asian‐American and ten as White	Semi‐structured Interviews Inductive Thematic analysis	There were different types of moral boundary‐work performed: the white women interviewed, primarily relied on biomedical evidence when making claims of legitimate disability; Black respondents often referenced trauma, abuse and discrimination, in addition to their diagnoses, to signal their deservingness of aid. All women described at least one instance of where the legitimacy of their pain was doubted
54 Råheim and Håland (2006). Lived Experience of Chronic Pain and Fibromyalgia: Women's Stories From Daily Life (Norway)	42	To describe and understand women's lived experience of chronic pain and fibromyalgia	12 women with Fibromyalgia Age 34–51 years White	Life‐form interviews Phenomenology	Three typologies 1. At the will of the treacherous body‐powerlessness (Morning—The Prison of the Body, Forenoon and Afternoon Needs) Against the Body and Giving Up, (Evening—Endless Pain, Feeling Useless and in Despair; Important Relations—Lack of Recognition and Support) 2. Struggling to escape the treacherous body‐ ambivalence (Morning—The Body “Threatens to Take Full Control”, Forenoon and Afternoon—Fighting With or Against the Body, Evening—Overwhelmed by Pain and Feeling Desperate; Important Relations—Recognition but Difficulty in Communicating Needs) 3. Caring for the treacherous body‐coping (Morning—Persuading an Unwilling Body, Forenoon and Afternoon—Forget About the Body on the Background of Taking Care, Evening—Pain, but Still Coping; Important Relations—Mutuality and Dialogue)
55 Reibel and Pearson (2017). Beyond the Pain: A Look into the Experiences of Women Living with Fibromyalgia (United States)	43	To gain an understanding of the lived experiences of women with fibromyalgia	3 females with fibromyalgia	In‐depth interviews (phenomenological study) Thematic Analysis	1. There is nothing we can do for you, 2. We've got to find something, 3. I feel like I'm going crazy, 4. losing, 5. the best day and 6. hope: I can do this
56 Rice et al. (2024). Gendered worlds of pain: Women, marginalization, and chronic pain (Canada)	69	The study aimed to explore the intersection of chronic pain, poverty and gender, focusing on how individuals with low socioeconomic status (SES) and chronic pain navigate the challenges posed by both	24 women (between their 20s and 70s) experiencing chronic pain and socioeconomic marginalization	In‐depth, semi‐structured interviews Indexing and Coding within an Institutional Ethnography Framework	Four key, interrelated themes: (1) gendered minimization of women's health concerns, (2) managing intergenerational poverty, (3) living with violence and trauma and (4) gendered organization of family care
57 Richardson (2005). Establishing the (extra)ordinary in chronic widespread pain (Australia)	44	To illustrate the ways in which the invisible, subjective and everyday nature of chronic pain leads to sufferers experiencing delegitimation of their condition	6 females with chronic widespread pain Age 51–88 years	Interviews Interpretive design	1. ‘They're called bludgers’: delegitimation of the pain 2. Extraordinary stories: legitimating chronic pain
58 Roberto and Reynolds (2002). Older women's experiences with chronic pain: Daily challenges and self‐care practices (United States)	45	To understand the influence of chronic pain on older women's lives	20 Females living with chronic pain Age 48–86 years	Focus groups (*n*‐20) In‐depth interviews (*n* = 8) Thematic Analysis	1. Pain has personal and shared meaning 2. Beliefs about pain are reinforced by societal stereotypes and the reactions of others 3. Formal interventions and informal coping strategies often are used in tandem to manage pain 4. living with chronic pain requires changes in daily activities and routines 5. Pain influences interactions with family members and friends 6. pain challenges one's sense of self
59 Schaefer (1995). Struggling to maintain balance: a study of women living with fibromyalgia (United States)	46	To describe the experiences of women living with fibromyalgia	36 Women with fibromyalgia Age range and ethnicity not identified	In‐depth interviews (*n* = 36) Follow‐up interviews (*n* = 6) A combination of Grounded theory and feminist methods	1. Struggling to maintain a balance Within this process includes recalling perceived normalcy, searching for a diagnosis, finding out and moving on. Several women's stories indicated that they gave up the struggle to maintain a balance: ‘relinquishing the struggle’
60 Skuladottir and Halldorsdottir (2011). The quest for well‐being: self‐identified needs of women in chronic pain (Iceland)	47	To study the self‐reported needs of women in chronic pain	5 women with chronic Pain Age 36–53 years	in‐depth interviews Phenomenology (the Vancouver School)	Three major quests: a quest to learn to live with pain by making it tolerable and a quest to find support from someone who cares, professional support and the need to be connected to others. Also, a quest for normalcy by trying to avoid the sick role and maintain a sense of dignity. The overriding theme in all the three major quests is the quest for well‐being: physical, mental, emotional and social
61 Söderberg and Lundman (2001). Transitions experienced by women with fibromyalgia (Sweden)	48	To illuminate the transitions experienced by women with Fibromyalgia	25 women with Fibromyalgia Age 35–60 years	Interviews Content Analysis	Transitions occur in different areas of the women's life—in daily life pattern, in working life, in social life, in family life—but participants expressed the view that they were learning to live with the changes. Fibromyalgia as choreographer of activities/relationships
62 Söderberg et al. (1999). Struggling for dignity: The meaning of women's experiences of living with fibromyalgia (Sweden)	49	To explore the meaning of women's experiences of living with fibromyalgia	14 women with Fibromyalgia Age 35–50 years	Interviews Phenomenology	Loss of freedom Threat to integrity A struggle to achieve relief and understanding
63 Wade and Shantall (2003). The meaning of chronic pain: A phenomenological analysis (South Africa)	50	To provide a description of the life‐world of people with chronic low back pain	Three females, who had experienced unrelieved, continuous chronic low back pain	Interview Phenomenology	Chronic pain is persistent, giving it quality that is particularly difficult to endure. Pain Takes Over Your Life Chronic Pain Causes Interpersonal Difficulties One is Alone Chronic Pain Creates a Bleak Future Coping with Chronic Pain Finding Meaning in Suffering
64 Werner and Malterud (2003). It is hard work behaving as a credible patient: Encounters between women with chronic pain and their doctors (Norway)	51	To explore women chronic pain patients' consultation experiences with their doctors and ‘work’ done by the patients in order to be believed, understood and taken seriously	10 women with chronic muscular pain Age 26–58 years Norwegian and one Asian immigrant	Semi‐structured in‐depth interviews Phenomenology	The women patients' accounts indicated hard work to make the symptoms socially visible, real and physical when consulting a doctor. Their efforts reflect a subtle balance not to appear too healthy or too sick
65 Werner et al. (2004). ‘I am not the kind of woman who complains of everything’: illness stories on self and shame in women with chronic pain (Norway)	52	To explore issues of self and shame in illness accounts from women with chronic pain	10 women with chronic muscular pain Age 26–58 years Norwegian and one Asian immigrant	In‐depth Interviews Phenomenological discourse analysis	Stories told about (positive) strength of the women living with pain and the (negative) illness talk of others. Talk of coping and credibility
66 Westergården et al. (2021). ‘Moving between living in the shadow of pain and living a life with the pain in the shadows’ – women's experiences of daily life with chronic widespread pain: A qualitative study (Sweden)	53	To explore women's experiences of the impact of living with CWP on daily life	19 women with chronic widespread pain (CWP) Age 45–67 years	Interviews Content Analysis	Moving between living in the shadow of pain and living a life with the pain in the shadows: (1) living with invisible challenges (feeling neglected as a person and feeling lonely among other people) (2) struggling with limitations (moving between ability and inability, stress and worries and being dependent on others) (3) encountering daily life with varying degrees of flexibility (standing still and giving up, moving back and forth by adapting and striving forward with resistance)
67 White and Seibold (2008). Walk a mile in my shoes: An auto‐ethnographic study (Australia)	54	To uncover and understand the reality of living with chronic, intractable, non‐malignant back pain from individuals living with it	5 females with chronic back pain Age 32–44 years	Auto‐ethnographic study Interviews and Journaling Thematic analysis	Key Themes: Loss of control, speaking into the void (the frustration experienced by participants when they felt they were not being listened to or heard by HCPs), body image disruption, attempting to find meaning and mourning the loss (loss of relationships), putting on a mask, being stigmatized and everyone knows best
68 Wong et al. (2023). Challenges, Concerns, and Experiences of Community‐Dwelling Older Women with Chronic Low Back Pain‐A Qualitative Study in Hong Kong, China (China)	70	To investigate the experiences, challenges, concerns, and coping strategies of older women with CLBP in Hong Kong	15 community‐dwelling older women with CLBP aged 60–90 years	Semi‐structured interviews Thematic Analysis	Five themes: (1) physical impacts of CLBP on daily life; (2) psychological influences of CLBP; (3) management of CLBP; (4) family support; and (5) social activities and support
69 Wuytack and Miller (2011). The lived experience of fibromyalgia in female patients, a phenomenological study (Belgium)	55	To gain a better understanding of the subjective experience of fibromyalgia, focusing on the personal, occupational and social impact of the condition on patients' lives	6 females with Fibromyalgia Age 36–66 years	Semi‐structured interviews (phenomenological study) Thematic analysis	Fibromyalgia pervaded all aspects of life. It impacted on the participants' (1) Occupation (2) personal life (3) Views about the future (4) Interaction, communication and expression
70 Zander et al. (2013). Struggling for sense of control: Everyday life with chronic pain for women of the Iraqi diaspora in Sweden (Sweden)	56	To understand the specific needs of women living with chronic pain from the Iraqi diaspora in Sweden	11 females 40–64 years of age (table), with chronic musculoskeletal pain	Semi‐structured interviews Grounded theory	1. Living as a migrant 2. The changing pain 3. Efforts to control pain 4. Mediating resources The women's everyday life was characterized by a struggle for a sense of control. The struggle was multi‐dimensional and influenced by their situation in life together with their unpredictable pain

There is no one method or technique for qualitative meta‐synthesis. A flexible and creative approach is encouraged; one which does not violate philosophical foundations and methodological assumptions, is systematic and is suited to the question of the project undertaken (Sandelowski & Barroso, 2007). Inductive reflexive thematic analysis (Braun & Clarke, 2022) was deemed appropriate for the aims of the review and it was adopted to analyse and synthesize the findings as this method can be used with a variety of qualitative paradigmatic approaches (Braun & Clarke, 2006, 2022; Kiger & Varpio, 2020). Themes were generated by analysing the abstracts, findings, discussions and conclusions of the included studies, following the guidelines provided by Braun and Clarke (2022).

Analysis began with data familiarization (reading the above‐mentioned text and highlighting the themes/participant quotes). During this stage, initial themes and participant quotes were highlighted to identify aspects relevant to the review question. Codes were then assigned to any data segment that focused on women's experiences of living with pain. This coding process was iterative, involving repeated engagement with the data to refine and adjust codes, ensuring they remained grounded in the original studies while addressing the meta‐synthesis aims.

Following the coding process, themes were developed from the finalized codes. Thematic maps were employed to explore relationships between themes, serving as a visual aid to ensure coherence and uncover connections across the dataset. This mapping process allowed for the identification of overlapping or closely related themes, which were subsequently grouped together and renamed to enhance clarity and relevance.

The iterative data analysis process involved revisiting the data and themes across studies for continuous refinement and deeper understanding. Peer debriefings further examined these themes, honouring the complexities of the participants' lived experiences, with the aim of ensuring the interpretations were anchored in the original findings. By revisiting the data and refining thematic structures, the process ensured that the final themes were meaningful and representative of the synthesized literature. Studies included from the updated search were thematically analysed (as above) and then integrated into the existing synthesis with a refinement of the final themes. The final themes were devised to encapsulate the complexity of the findings and provide insights into the broader implications of the research.

For the narrative write‐up, data excerpts that best exemplified the final themes were carefully selected. Some of these and additional ones were used to support the GRADE‐CERQual evaluation (see Tables 5 and 6). The final narrative synthesis not only provided a cohesive account of the results but also highlighted key gaps in the existing literature, offering opportunities for future research and advancing understanding of chronic pain experiences.

**TABLE 5:** GRADE‐CERQual evaluation.

Finding	Studies contributing to the theme	Assessment of methodological limitations	Assessment of relevance	Assessment of coherence (1)	Adequacy	Overall assessment/explanation
Pain and multiple responsibilities Across the studies, women consistently reported that their lives were profoundly shaped by the dual burden of chronic pain and multiple responsibilities. Pain emerged as a central determinant, governing their capacity to engage in or abstain from various activities. For many, the demands of their responsibilities not only heightened their sense of strain but also exacerbated their physical discomfort, creating a cyclical relationship wherein their obligations exacerbated their pain	1, 2, 3, 4, 5, 6, 8, 10, 11, 13, 14, 15, 16, 17, 18, 19, 21, 24, 25, 27, 29, 30, 31, 35, 36, 37, 39, 40, 42, 44, 45, 47, 48, 50, 52, 53, 54, 55, 56, 57, 58, 59, 60, 61, 62, 63, 64, 65, 66, 67, 68, 69, 70	No/minor concerns (all studies were rated as ‘++/+’ in this review)	Minor concerns. All women over the age of eighteen, living with chronic pain in any setting was specified in the review question. All the studies satisfied these criteria and focused on their experiences. Various chronic pain conditions/countries represented (17) countries; (Portugal not included)	No/minor concerns about coherence	Minor concerns Findings evaluated as rich across 41 studies 53 contributing studies	High confidence due to there being only minor concerns across all domains. Fifty‐three studies contributing and the finding is described richly across 41 of the contributing studies
Countless Losses (and their psychological effects) Many women described enduring a multitude of losses, including their physical functionality, sleep, confidence, sense of self, future aspirations, dreams, goals, employment opportunities, and personal relationships. These profound losses significantly impacted their psychological well‐being, deepening the challenges they faced and underscoring the pervasive effects of pain/the losses on various aspects of their lives.	2, 3, 6, 8, 10, 11, 13, 14, 16, 17, 18, 19, 20, 21, 22, 24, 25, 27, 28, 29, 30, 31, 32, 33, 34, 35, 36, 37, 38, 39, 41, 42, 43, 44, 45, 46, 48, 49, 50, 51, 52, 53, 54, 55, 56, 57, 58, 59, 60, 61, 62, 63, 64, 65, 66, 67, 68, 69, 70	No/minor concerns (all studies were rated as ‘++/+’ in this review)	No major concerns (as above) Countries represented: 16 (not included: Portugal and Iceland)	No/minor concerns	Minor concerns Findings evaluated as rich across 47 studies Contributing (59)	High confidence due to no/minor concerns across all four domains. The findings are broadly relevant to women's experiences with pain across diverse populations. What might vary is the nuance or amplification of these themes in underrepresented groups, such as women from ethnic minority populations. This is discussed. Many studies contributing (59) to the review finding and richly described in 47
Lack of Understanding: Delegitimizing and Disempowering Encounters Many women reported feeling misunderstood by those around them, including HCPs. They described experiencing various disempowering interactions and expressed frustration at the lack of adequate information and guidance provided to help them manage their pain effectively. These challenges contributed to a sense of isolation and hindered their ability to navigate their condition with confidence.	1, 2, 3, 4, 5, 6,7, 8, 9, 10, 11, 12, 13, 14, 17, 18, 19, 20, 22, 23, 25, 26, 28, 30, 31, 32, 33, 34, 36, 37, 38, 39, 40, 41, 42, 43, 44, 45, 46, 47, 48, 49, 50, 51, 52, 53, 54, 55, 57, 58, 59, 60, 61, 62, 63, 64, 65, 66, 67, 68, 69, 70	No/minor concerns (all studies were rated as ‘++/+’ in this review)	As above Countries represented: 17 (not included: Chile)		Findings evaluated as rich across 47 studies Studies contributing (62)	High confidence due to no/minor concerns across all four domains. However, the experience of women from different ethnic backgrounds may amplify the intensity of feeling misunderstood due to factors such as language differences, cultural stigma, or experiences of bias (discussed). Many studies contributing (62) to the review finding and richly described in 47
Solace and Self‐empowerment Many women, after receiving limited information, sought solace and self‐empowerment by actively seeking help and information elsewhere. They engaged in support groups, participated in activities that fostered mental well‐being, and took proactive steps to better understand and manage their situations, striving to regain a sense of control over their lives.	1, 2, 3, 4, 5, 6, 7, 8, 9, 10, 11, 12, 13, 15, 16, 17, 18, 19, 22, 23, 24, 25, 26, 27, 29, 31, 32, 34, 39, 40, 42, 43, 44, 45, 46, 47, 48, 49, 50, 51, 54, 55, 56, 57, 58, 59, 60, 61, 62, 63, 64, 65, 66, 67, 69, 70	No/minor concerns (all studies were rated as ‘++/+’ in this review)	As above no concerns 17 countries represented (Denmark not included)		Findings evaluated as rich across 44 studies Studies contributing (56)	High confidence due to no/minor concerns across all four domains. As mentioned above, seeking solace could take culturally specific forms, such as community‐based coping mechanisms or traditional practices. Many of the studies contributing (56) to the review finding and richly described in 44

*Note*: List of studies (with their GRADE‐CERQual ID numbers) can be found in Table 4. (1) Coherence (a measure of how clear and well supported the data from the primary studies and a review finding are: consistency across studies), adequacy of data (a measure of how rich and well supported a review finding is by the included studies) and relevance (how well the evidence from the primary studies fits into the context of the review question).

**TABLE 6:** Studies contributing to each theme.

Study authors (date)	Pain and multiple responsibilities	Countless losses (and their psychological effects)	Lack of understanding: delegitimizing and disempowering encounters	Solace and self‐empowerment
Ahlsen et al. (2014)	x		x	x
2 Altun et al. (2023)	x	x	x	x
3 Allen et al. (2015)	x	x	x	x
4 Arman et al. (2020)	x	x	x	x
5 Barnes et al. (2021)	x		x	x
6 Bostick et al. (2018)	x		x	x
7 Campbell et al. (2022)	x	x	x	x
8 Campeau (2018)			x	x
9 Caton et al. (2024)	x	x	x	x
10 Dickson and Kim (2003)	x	x	x	x
11 Driscoll et al. (2018)			x	x
12 Dysvik et al. (2013)	x	x	x	x
13 Evans and de Souza (2008)	x	x	x	x
14 Gonzalez et al. (2015)			x	x
15 Gullacksen and Lidbeck (2004)	x	x	x	x
16 Hallberg and Carlsson (1998)	x	x	x	
17 Hallberg and Carlsson (2000)	x			x
18 Hervik et al. (2023)	x	x	x	x
19 Horment‐Lara et al. (2022)	x	x		x
20 Howell (1994)	x	x	x	x
21 Hwang et al. (2004)	x	x	x	x
22 Ito and Pascual (2024)	x	x	x	x
23 Johnson et al. (2024)	x	x	x	x
24 Juuso et al. (2011)	x	x	x	x
25 Juuso et al. (2014)		x	x	
26 Juuso et al. (2016)	x	x		
27 Kanter et al. (2017)		x	x	x
28 Kengen Traska et al. (2012)			x	x
29 Kirkham et al. (2015)	x	x		x
30 Knutsen et al. (2022)	x	x	x	x
31 Lehti et al. (2017)			x	x
32 Lightbourne et al. (2024)	x	x	x	x
33 Löfgren et al. (2006)	x	x		x
34 Lo Monaco et al. (2024)	x	x	x	x
35 Mellado et al. (2016)		x	x	
36 Mellado et al. (2020)	x	x		x
37 Meriwether et al. (2022)	x	x	x	x
38 Michaëlis et al. (2015)	x	x	x	
39 Molin et al. (2021)	x	x	x	x
40 Molin et al. (2022)		x	x	x
41 Molin et al. (2024)	x	x	x	x
42 Monsivais (2013)		x	x	
43 Müllersdorf et al. (2011)		x	x	x
44 Mustafa et al. (2020)	x	x		
45 Mustafa et al. (2024)	x	x	x	x
46 Nortvedt et al. (2015)	x	x	x	
47 Nortvedt et al. (2016)	x	x	x	
48 Nyen and Tveit (2018)		x	x	
49 Osborn and Smith (1998)	x	x	x	x
50 Park et al. (2022)	x	x	x	x
51 Peppard et al. (2022)	x		x	x
52 Peterson et al. (2023)	x	x	x	
53 Pryma (2017)		x	x	
54 Råheim and Håland (2006)	x	x	x	x
55 Reibel and Pearson (2017)		x	x	x
56 Rice et al. (2024)	x	x	x	x
57 Richardson (2005)	x	x	x	x
58 Roberto and Reynolds (2002)	x	x	x	x
59 Schaefer (1995)		x	x	x
60 Skuladottir and Halldorsdottir (2011)	x		x	x
61 Söderberg and Lundman (2001)	x	x	x	x
62 Söderberg et al. (1999)		x	x	x
63 Wade and Shantall (2003)	x	x	x	x
64 Werner and Malterud (2003)		x	x	x
65 Werner et al. (2004)	x	x	x	
66 Westergården et al. (2021)	x	x	x	
67 White and Seibold (2008)	x	x	x	x
68 Wong et al. (2023)	x	x	x	x
69 Wuytack and Miller (2011)	x	x	x	x
70 Zander et al. (2013)	x	x		x

## RESULTS

### Study characteristics

The seventy studies included in the review were published between the years 1994 and 2024 and were conducted in eighteen countries: Australia (*n* = 4), Belgium (*n* = 1), Brazil (*n* = 2), Canada (*n* = 7), Chile (*n* = 2), China (*n* = 1), Denmark (*n* = 1), England (*n* = 2), Iceland (*n* = 1), Ireland (*n* = 1), Italy (*n* = 1), Korea (*n* = 2), New Zealand (*n* = 1), Norway (*n* = 10), Portugal (*n* = 1), South Africa (*n* = 1), Sweden (*n* = 17) and the United States (*n* = 15). Twenty‐five studies focused on chronic pain in general (across a range of conditions), while others focused on specific conditions: fibromyalgia (*n* = 16), chronic neck pain (*n* = 1), chronic low back/back pain (*n* = *5*), rheumatoid arthritis (*n* = 1), osteoarthritis (*n* = 1), MSK pain (*n* = 5), interstitial cystitis/bladder pain syndrome (*n* = 1), pelvic girdle pain (*n* = 1), chronic pain after childbirth (*n* = 1), endometriosis and chronic pelvic pain (*n* = 4), chronic pelvic pain (*n* = 3), chronic widespread pain (*n* = 2), chronic muscular pain (*n* = 2), myofascial pain syndrome and fibromyalgia (*n* = 1) and chronic headaches (*n* = 1). The twenty‐five studies that focused on chronic pain in general included women with a range of conditions: lupus, multiple sclerosis, Ehlers‐Danlos syndrome, irritable bowel syndrome, migraines, repetitive strain injury and phantom limb pain. However, in some of these studies, the number of women with each condition was not always clear. Various data collection methods were used, including interviews, focus groups, observations, written narratives, reflective photo voice and online qualitative surveys. Various approaches to data analysis were also adopted, including phenomenology, thematic analysis, content analysis and grounded theory. The sample size of the studies included in this review ranged from three to five hundred and thirty‐two women. The total number of women from the seventy studies was one thousand five hundred and seventy. The ages of the participants ranged from eighteen to ninety years. The ethnicity of the participants was not identified in many studies (see Table 4 for details).

### Study quality

Following an evaluation of the included studies' methodological limitations, fifty‐two were rated as “++” (all or most of the criteria of the checklist had been met) and eighteen as “+” (some of the criteria had been met, but it was deemed unlikely to affect the conclusion; see Table 3).

### Themes

Four themes were generated following thematic analysis: Pain and Multiple Responsibilities; Countless Losses (and Their Psychological Effects); Lack of Understanding: Delegitimizing and Disempowering Encounters; and Solace and Self‐Empowerment. Confidence in all themes was rated as high (see Tables 5 and 6 for GRADE‐CERQual Evaluation).

### Pain and multiple responsibilities

This theme captures the dual burden faced by women in navigating the pervasive influence of chronic pain alongside the demands of their daily lives. Women in the studies expressed that their lives revolved around their pain and that they also had numerous other responsibilities to deal with: “The pain takes over… there is nothing except pain and work” (Hallberg & Carlsson, 1998). Pain impacted all areas of their lives and was described as a “constant”. Participants reported experiencing it “every day” (Molin et al., 2024) and it prevented them from engaging in activities that they enjoyed (Johnson et al., 2024). Another participant stated how it impacted “everything in everyday life. It limits me in all my roles as a mother, wife, cook, friend… put a stop to ambitions and goals” (Hervik et al., 2023).

Many also discussed the strain of balancing multiple other responsibilities with their pain and the additional burden this created (Johnson et al., 2024). These responsibilities consisted of employment outside the home, housekeeping, shopping, parenting and caregiving. The latter were often exacerbating their pain (Rice et al., 2024). Parenting and pain were described as a “dual burden” for mothers living with chronic pain. A participant stated that “looking after her children was the hardest part of pain” (Evans & de Souza, 2008). Women were also mostly responsible for housework (even if they were in pain and unable to work outside the home; Richardson, 2005).

For some women there was “never a day without pain” (Hervik et al., 2023). Women spoke of how each day was dictated by their pain: “You can't just do anything you want. You've now got this little thing called severe back pain constantly holding you in check” (White & Seibold, 2008). Another stated, “In any situation… my back comes first. Every situation you can possibly think of, that's what my back stops me from doing” (Evans & de Souza, 2008). Women often expressed taking it “one day at a time” (Hallberg & Carlsson, 1998) and not being able to plan for the future (Roberto & Reynolds, 2002). Another participant stated, “My life actually revolves around my pain most of the time… it is something I've had to come to terms with and live with” (White & Seibold, 2008). Younger and older women felt this restrictive nature of pain controlling their lives, as well as an inability to plan for the future and participate fully in life. The pain was not only a barrier to physical activity but also a hindrance to independence and self‐identity. Women described feeling “trapped,” some in their homes, some in their situations and some by negative thoughts (Hervik et al., 2023; Park et al., 2022; Peterson et al., 2023). Pain was central to their lives, shaping their daily experiences, actions and thoughts.

Confidence in this theme was evaluated as high. Of the seventy studies included in this review, fifty‐three contributed to this theme (see Table 5). There were very few minor concerns regarding methodological limitations. The theme was evaluated as richly described across forty‐one studies and represented across seventeen of the eighteen countries identified in the review.

### Countless losses (and their psychological effects)

This theme was strongly represented in the experiences reported by many women living with chronic pain. Many women described enduring a multitude of losses, including their physical functionality, autonomy, sleep, confidence, work, sense of self, self‐esteem, identity, future aspirations, dreams, goals, their former lives, employment opportunities, hope and personal relationships (Hervik et al., 2023; Ito & Pascual, 2024; Lightbourne et al., 2024; Peterson et al., 2023).

For some, these losses were deeply distressing, and the feeling of losing themselves was profoundly unsettling: “I lost myself, somewhere” (Hervik et al., 2023). Another spoke of the loss of her identity that had been “washed away by pain and illness” (Hervik et al., 2023). “The pain has stolen from me the roles of mother, wife, friend, and work colleague” (Dysvik et al., 2013). These sentiments capture the significant impact that chronic pain can have on a person's self‐concept and social roles. Another participant described the loss of both her work and identity: “I had to quit working and that was just incredibly horrible for me because my identity has always been very tied up with my profession” (Howell, 1994), and another participant described the impact on everyday life: “You can't go shopping or go for a walk with the baby in a pram” (Knutsen et al., 2022).

The loss of the life they had hoped for was also experienced and expressed: “I'm only 50 and I should be doing this that and the other cos they say life begins at 40 but I can't and I s'pose it does bother me, it's frustrating that people of my own age are…and you feel as if you can't” (Osborn & Smith, 1998). The loss of functioning, control and relationships echoed across the studies. A woman stated that she found it extremely challenging to “not to be in control of myself” (Knutsen et al., 2022). These losses were accompanied by negative feelings, beliefs and painful emotions: grief, loneliness, sorrow, anguish, anxiety and sadness: “You want to be able to do what you were able to before—it creates a lot of anxiety and grief” (Arman et al., 2020). These losses impacted every aspect of their lives and exacerbated their well‐being (Ryff, 1989).

Fifty‐nine studies contributed to this theme (see Table 5). There were very few minor concerns regarding methodological limitations. The theme was found to be comprehensively described in forty‐seven studies. It was identified across sixteen of the eighteen nations identified in the present review, and confidence in this theme was evaluated as high.

### Lack of understanding: Delegitimizing and disempowering encounters

Women reported how they felt that others did not understand their conditions, predicaments, or the challenges that they faced. They stated that this was due to their pain not being visible (Hervik et al., 2023; both physically and clinically): “They don't understand it [chronic pain] because they don't see it. I used to give a lot of explanation, but now I can't be bothered anymore. There are very few people who understand it” (Wuytack & Miller, 2011). Women stated that they experienced stigmatization from others and from health care professionals (HCPs) because of this lack of understanding and visibility. Women across the studies reported feeling “dismissed,” “rejected,” “disbelieved” and “ignored” by HCPs. Women reported being met with disbelief and discouragement. A woman spoke of how her neurologist dismissed her condition (fibromyalgia): “There are believers and non‐believers, and I am a nonbeliever, so we're not going to talk about that” (Wuytack & Miller, 2011). This was also echoed by another participant: “The rheumatologist that I saw knew what fibromyalgia was, but he didn't believe in it” (Reibel & Pearson, 2017). Another participant stated that “unless you have a huge gaping wound or something that they can see, it's really hard to be treated for pain” (Campbell et al., 2022; see also Werner & Malterud, 2003).

These encounters are evident throughout the literature, with both older studies and more recent research illustrating them. In a very recent study, a participant reported how she felt she was “treated like an idiot” by HCPs. These encounters led many women to give up seeking further help (Molin et al., 2024). Another stated that she was told that “women often exaggerate pain more than men” (Johnson et al., 2024). There was a clear loss of trust in HCPs, as one woman commented that “you have to find your own way” (Molin et al., 2024). Participants reported feeling that HCPs “don't really understand… how debilitating the pain can be” (Johnson et al., 2024). “The provider thought that I was exaggerating about the pain” (Park et al., 2022). One participant noted, “a lot of doctors said that it was in my head” (Caton et al., 2024). The general sentiment was that there were gaps in the knowledge of HCPs regarding understanding and managing pain. Some openly stated that HCPs should “be more knowledgeable or educated about it [pain]” (Molin et al., 2024). Others found it difficult not to be understood by their family and deeply desired for their family to understand their pain the most (Lo Monaco et al., 2024).

Furthermore, some of the women felt that they also lacked knowledge about their condition because of their HCP's lack of knowledge and the incorrect information provided to them. Women also felt that their pain was trivialized and not taken seriously: “It's just not taken seriously [chronic pain]. I think it's very difficult for women to be heard” (Bostick et al., 2018). Other women were told to “go home and take two aspirins” or that the pain was in their minds (Roberto & Reynolds, 2002). Women had hopes and expectations when they sought health care. However, very few women reported leaving a consultation feeling hopeful, empowered and validated. Many women left with all their hopes of a diagnosis and effective treatment destroyed.

The present theme was found to be comprehensively described in forty‐seven of the sixty‐two contributing studies (see Table 5). There were only minor concerns regarding methodological limitations. The theme was identified across seventeen countries identified in the present review, and confidence in it was evaluated as high.

### Solace and self‐empowerment

Many of the women in this study were seeking solace and self‐empowerment and were trying to self‐manage, ease their pain and make their lives as comfortable as they could. They were actively seeking knowledge about their condition, and some had researched extensively: “You have to become your own doctor, I swear. You've got the three years medical school, but you don't have the certificate hanging on your wall” (Campbell et al., 2022). Many women continued on their quest for self‐empowerment, and they remained hopeful: “I don't rely on anybody. This is absolutely my lone battle against the disease. I have to overcome it by myself” (Hwang et al., 2004); they did not want to be a “burden” to others (Horment‐Lara et al., 2022).

Some women sought solace in religion, and others gained strength from a belief in a “higher force” (Gonzalez et al., 2015). Others took up new hobbies as a way of regaining a purpose in life and engaged in meaningful activities (volunteering and helping others; Hwang et al., 2004; Wuytack & Miller, 2011). Many women sought solace by joining support groups and engaging in activities that distracted them from pain, providing opportunities for social relationships and personal development (Allen et al., 2015; Lo Monaco et al., 2024). They also sought advice from the internet and social media and turned to “God for strength” (Mellado et al., 2020; Molin et al., 2022), which helped them to also remain hopeful. Some participants found solace in baking, other social activities, online communities or support groups where they could share their stories and find understanding and solidarity from others in similar situations (Hervik et al., 2023; Meriwether et al., 2022; Rice et al., 2024; Wong et al., 2023). Others sought comfort in maladaptive coping mechanisms to deal with their low mood, mentioning alcohol consumption (Caton et al., 2024). This highlights the real need for psychosocial interventions to address these low moods, which will also influence how individuals experience pain.

This theme was acknowledged across seventeen countries identified in the present review, and confidence in it was evaluated as high (see Table 5). There were only minor methodological concerns, and the theme was comprehensively described across forty‐four of the fifty‐six contributing studies.

## DISCUSSION

This meta‐synthesis aimed to explore, evaluate and analyse women's experiences of living with chronic pain. The first theme highlighted the challenges women face in managing chronic pain while fulfilling their daily responsibilities. Their lives were dominated by pain, described as a constant presence that affected all areas of their lives, limiting their roles and aspirations. The relentless dominance of pain, compounded by multiple responsibilities, creates significant stress and, for some, inhibits them from seeking treatment. Domestic work is a strongly gendered activity, with women shouldering a greater share of household responsibilities compared to men (Harryson et al., 2012). This has implications for the support provided to assist women living with chronic pain. The impact of cultural expectations and domestic responsibilities fits within the biopsychosocial model (Engel, 1977), which underscores the importance of considering both psychological and social factors in the management of chronic pain. HCPs, policymakers and those supporting women with chronic pain need to be cognizant of the additional burden posed by domestic and caregiving responsibilities. These expectations, often gendered and culturally ingrained, can exacerbate the experience of pain through increased stress, reduced rest and limited opportunities for self‐care. As such, efforts should be made to reduce these burdens and provide appropriate support to alleviate their impact.

Strategies may include promoting equitable sharing of domestic tasks within households, offering education to family members about the impact of chronic pain and integrating practical supports such as access to respite care, domestic assistance, or subsidized services (e.g., cleaning, grocery delivery, or prepared meal options). Time‐saving strategies such as batch cooking, simplified meal planning, or utilizing community kitchens can also help reduce the physical load of daily food preparation. HCPs can play a pivotal role by initiating conversations around role strain, connecting women with community‐based resources and encouraging the involvement of supportive family/friends in pain management plans. Beyond this, employers and policymakers should explore inclusive workplace practices that enable flexibility for women with chronic pain, including remote work options.

Other actionable steps may include HCPs providing direct referrals to patient‐led or professionally facilitated groups, either in person or online. Support helps individuals deal with stressors. According to the stress‐buffering hypothesis, social support functions as a protective factor that mitigates the detrimental effects of stress on health outcomes (Cohen & Wills, 1985; Meints & Edwards, 2018). Social support has been linked to enhanced physical functioning among individuals living with pain‐related conditions, buffering the negative effects of pain‐related stress, helping prevent feelings of isolation and reducing the burden of everyday tasks (Centre for Pain Management, 2024; Che et al., 2018; Meints & Edwards, 2018).

In addition to this, interventions that focus on distraction techniques could also be explored as a means to alleviate the overwhelming dominance of pain in individuals' lives (Ambron, 2022; Mansell et al., 2020; Tabibnia, 2020). Distraction techniques, such as engaging in enjoyable activities, help reduce the dominance of pain by diverting attention away from discomfort. This concept is rooted in the idea that by redirecting focus, individuals can reduce the emotional intensity associated with pain, thus improving overall well‐being. Distraction strategies reflect cognitive‐behavioural models of pain, where shifting attention away from pain can lessen its perceived intensity and emotional impact.

The second theme underscored the countless losses women experienced due to chronic pain, including physical functionality, identity, aspirations, relationships and independence, profoundly affecting all six dimensions of psychological well‐being (Ryff, 1989). Many of the participants compared their current reality to where they wished or hoped to be, revealing a clear discrepancy that would also impact their psychological well‐being (Higgins, 1987; Osborn & Smith, 1998).

Furthermore, women reported loss of relationships and experiencing loneliness. It is important to address the latter, as loneliness undermines psychological well‐being and has been connected to various mental health problems, suicide, addiction and depression (Russell et al., 1984). Loneliness is both physically and emotionally painful, and it is a form of stress (Hainer, 2012). This can exacerbate pain, and stress also decreases an individual's tolerance for pain (Ahmad & Zakaria, 2015; Lang, 2020; Melzack, 2001). Thus, pain management interventions should be devised to address both. Support groups were found to be beneficial for women experiencing chronic pain in this review (Allen et al., 2015; Wong et al., 2023).

Significant losses were also reported by the participants with the loss of employment. Women expressed sadness at losing their job and being unable to work. Some referred to this loss as being associated with their identity. Job loss profoundly impacts psychological well‐being (The Mental Health Foundation, 2021). It can cause immense stress and anxiety, impact the self‐concept and cause depression, all of which also physiologically strain the body (British Psychological Society, 2019; Climent‐Rodríguez et al., 2019; Guindon & Smith, 2002; Jahoda, 1982; Jahoda et al., 2017; Paul & Moser, 2009) and further exacerbate pain. Loss of work has been described as “one of the most painful and traumatic events” an individual can experience (Climent‐Rodríguez et al., 2019) and one that involves them going through a process of grieving (Climent‐Rodríguez et al., 2019; Guindon & Smith, 2002). It has been stated that it is the loss of latent benefits (e.g., participation in collective goals and regular activity) associated with work that results in poorer psychological well‐being (Jahoda et al., 2017). An effort should be made to mitigate the existential losses experienced. Interventions should aim to provide a means of replacing some of these latent benefits and other losses. Some of the women in this review had already acknowledged this and had started to take up new hobbies as a way of regaining a purpose in life and engaging in meaningful activities.

The third theme directly highlights critical gaps in health care practice. The findings from these studies reveal deeply concerning patterns in how women living with pain experience interactions with HCPs. Recurrent reports of dismissal, lack of understanding and scepticism towards women's reported pain highlight significant gaps in the quality of care and empathy provided by HCPs.

Women frequently reported feeling belittled or dismissed by HCPs, as seen in remarks such as being “treated like an idiot” or accused of “exaggerating pain”. Such experiences not only invalidate the lived reality of their pain but also erode trust in health care systems. For many, this mistrust had culminated in disengagement from seeking further help. The cumulative effect of these interactions leaves women feeling abandoned in their pursuit of relief and understanding, as encapsulated in the sentiment, “you have to find your own way” (Molin et al., 2024). The review also revealed that while most experiences with HCPs were dismissive and disempowering, rare positive interactions, marked by effective listening and responsiveness, helped establish trust and encouraged help‐seeking. These valued qualities should be a focus for HCPs to improve patient care.

Another particularly troubling issue identified was the obvious gendered bias underlying some HCPs' responses, as reflected in the assertion that “women often exaggerate pain more than men” (Johnson et al., 2024). Such biases perpetuate stereotypes and contribute to a systematic underestimation of women's pain. Participants noted that HCPs often failed to grasp the debilitating nature of their pain, with comments like “the provider thought that I was exaggerating about the pain” (Park et al., 2022) and “a lot of doctors said that it was in my head” (Caton et al., 2024). These dismissive attitudes reflect a broader gap in HCPs' knowledge and training regarding chronic pain, particularly as it pertains to women.

Participants expressed a strong desire for HCPs to develop a deeper understanding of pain and its impacts. Suggestions for improved education and training were common, as was the expectation that HCPs should be better equipped to provide compassionate care. As one participant urged, HCPs should “be more knowledgeable or educated about it [pain]” (Molin et al., 2024). This aligns with broader calls for systemic reforms in medical training to prioritize the study of pain and its gendered dimensions. The lack of understanding extended beyond their interactions with HCPs, affecting their relationships with family members. Some participants expressed a profound longing for empathy and understanding from their families, highlighting the emotional toll of feeling misunderstood not only by professionals but also within their closest relationships. This underscores the need for broader societal awareness of pain and its impact on women's lives.

Chronic pain is a condition that is poorly understood and cannot be objectively validated and measured. It has been stated that the stigmatization potential of such conditions can increase because of this (Åsbring & Närvänen, 2002). Improving the public's and HCPs' understanding of chronic pain by educating them about women's experiences with chronic pain could be the key to raising awareness, compassion and reducing stigma (Carr, 2016; Nehrke et al., 2017; Stenhoff et al., 2015), especially for HCPs.

The studies included in this review demonstrated the power and influence of others on women's experiences of pain. HCPs have the power to influence the length and severity of women's pain. Medicine can be both a supportive institution that addresses or alleviates numerous problems and, at the same time, a restrictive one that may create or worsen other challenges (Howell, 1994; Williams, 2004). The review findings illustrated that patients feel HCPs are still guilty of stigmatizing women, resulting in the denial of treatment.

Women in the included studies were also dissatisfied with the quality and lack of information provided to them; this is especially important as communication and access to information are essential for empowering individuals to manage their condition (Molin et al., 2022). The former is even more crucial in the case of chronic conditions, as individuals must not only deal with a range of distressing symptoms but also learn to adjust to new and more restricted lifestyles. Failing to provide women with adequate information undermines their self‐efficacy and can shape negative beliefs about their condition (Bandura, 1982; Leventhal et al., 2016; Paterick et al., 2017). Self‐efficacy is critical for self‐management, as it directly influences behaviour, motivation and the approach to health‐related goals and challenges (Deci & Ryan, 1985; Paterick et al., 2017). Undermining an individual's sense of it not only denies effective treatment but also erodes their confidence in managing their condition, which can lead to maladaptive coping strategies like avoidance or resignation (Bandura, 1982; Leventhal et al., 2016).

Moreover, to not acknowledge an individual's account of their pain is to destroy all hope of managing it; if they are not believed, then they cannot be helped. Pain is a subjective experience; there is no gold standard test or measurement for it. Moreover, individuals might show their suffering in a variety of ways. It would thus be unethical to acknowledge the suffering of one person or group while ignoring the suffering of another. Furthermore, not all women may possess the mindset, amount of motivation, self‐efficacy and resilience necessary to transform these bad experiences into a desire for empowerment. Thus, HCPs need to be mindful of the power of their words and their positions.

The concept of self‐efficacy also intersects with other themes identified in the review. From the perspective of self‐determination theory (Deci & Ryan, 1985), fostering a sense of autonomy, competence and relatedness is essential for promoting sustained engagement in pain management strategies. When individuals feel empowered to make choices, believe in their capacity to manage pain (competence) and experience support from others (relatedness), they are more likely to adopt and maintain health‐enhancing behaviours. This also aligns with Bandura's Social Learning Theory: observing others successfully manage pain can enhance self‐efficacy by reinforcing the belief that one can achieve similar outcomes. Furthermore, consistent social support can reinforce self‐efficacy by providing models of effective coping, encouraging autonomy and offering validation (Bandura, 1982; Deci & Ryan, 1985). Similarly, feeling believed and understood by HCPs or close others can counteract feelings of helplessness, further promoting a sense of competence and control. Therefore, interventions that enhance social support, validation and shared learning may strengthen self‐efficacy and, in turn, improve pain management outcomes (Bandura, 1982; Deci & Ryan, 1985; Jackson et al., 2014).

Building on this, co‐designing care plans with women, grounded in their lived experiences and self‐identified needs, can further support self‐efficacy by fostering trust and positioning them as active agents in their care. Mindset change and cognitive reframing are useful strategies for women to manage pain more effectively by focusing on what they can do instead of what they cannot, thus fostering a sense of agency and resilience. Health services should implement culturally responsive care models that consider language barriers, cultural beliefs and historical health care discrimination to ensure more inclusive and equitable support for all women with chronic pain.

Addressing these issues requires not only improved medical education and training to reduce gender biases and knowledge gaps but also fostering a health care culture that prioritizes empathy and patient‐centred care. Furthermore, raising awareness within families and communities is essential to ensure that women living with pain feel supported and understood in all aspects of their lives. These findings also highlight the necessity of considering chronic pain management interventions within a biopsychosocial‐spiritual framework (Adams et al., 2006; Siddall et al., 2015; Sulmasy, 2002).

The final theme highlights how women sought solace and empowerment to bridge the gap left by insufficient support from their HCPs. The findings demonstrated that women were developing their own coping strategies at the individual and community levels; these must serve as one of the foundations for chronic pain management. Women's experiences and needs should be considered in their treatment and care, in devising interventions and to empower these women to live well with their conditions (Olshansky et al., 2005). A group where participants could meet to engage in activities they enjoyed, such as board games, singing and exercise, helped improve their mood, reduce stress and expand their social circle (Wong et al., 2023). This illustrates the importance of creating supportive environments that allow women to “regain that positive feeling” (Hervik et al., 2023), connection, purpose and relief from pain, emphasizing the need for holistic approaches to pain management that incorporate physical, psychological and spiritual aspects. There is a need for such interventions, as some will adopt unhelpful, maladaptive coping mechanisms. This need is underscored by participants who isolate themselves when their mood is low (Peterson et al., 2023) and others who turn to maladaptive strategies like alcohol consumption (Caton et al., 2024).

While the women in the review were able to turn to more accessible forms of care, such as self‐education or alternative methods (Campeau, 2018), little is known about the women who may face barriers like limited access to resources (whether due to language challenges, digital disparities, or a lack of technological literacy). Some women also spoke of cultural constraints, expectations and the level of freedom they had (Altun et al., 2023; Mustafa et al., 2020). These factors impact help‐seeking behaviours, highlighting the need for more accessible pain management interventions. The needs of women from different ethnic minority backgrounds are important areas to explore. Additionally, many women shared experiences of violent pasts and trauma. They reported feeling trapped in a cycle of recurring thoughts, repeatedly reliving traumatic experiences. Interventions should be designed with an understanding of patients' past traumas, ensuring that care is trauma‐informed and sensitive to the complexities of their individual experiences (Park et al., 2022; Rice et al., 2024).

The findings presented draw on the collective experiences and perspectives of women across seventy methodologically sound qualitative studies conducted in eighteen different countries. It presents four actionable dimensions and areas to address that could help improve pain management. This review identifies several potential actionable opportunities to improve care for women with chronic pain. Validating women's pain, increasing awareness, providing quality resources that meet the women's needs, better training for HCPs, developing tailored interventions aimed at restoring the losses and addressing various psychological well‐being dimensions and fostering community and family support are critical areas of focus. Addressing these gaps can lead to more equitable and effective pain management solutions.

### Strengths and limitations

A key strength of this review is its inclusion of a large number of studies and the diverse range of women's experiences it captures, combined with the rigorous application of the GRADE‐CERQual approach. It should be noted, however, that while the themes remain broadly relevant and confidence was assessed as high across all themes, the underrepresentation of certain groups of women highlights a limitation in the representation of the diversity of women's experiences. What may change is the amplification or intensity of these themes for underrepresented groups. For example, Mustafa et al. (2020) discussed how cultural expectations and associated responsibilities contributed to self‐neglect and overwork, which in turn intensified the experience of chronic pain among participants in their study. Lack of understanding might be amplified by language barriers, cultural stigma, or discrimination within health care systems (Bull et al., 2023; Lyman, 2021). Similarly, losses brought on by pain may involve additional layers of social or economic marginalization unique to certain groups. Finally, seeking solace could take culturally specific forms, such as community‐based coping mechanisms or traditional practices (Hastie et al., 2005).

The search strategy was planned to capture as many studies as possible. However, this was limited to studies published in English and in journals. Thus, the experiences of non‐English speaking women (that were not translated) and those not appearing in journals were not analysed. Also, due to the interpretive nature of this analysis, multiple interpretations are possible. However, the systematic nature of the analysis, adopting a reflexive approach and validation by all researchers aimed to ensure the review findings were representative of the participants' experiences in the studies (Braun & Clarke, 2022; Buetow, 2019; Dodgson, 2019).

## CONCLUSION

The themes that emerge from women's collective experiences outline a pathway of actionable steps: validating pain, increasing awareness, providing quality resources, improving HCP training, developing tailored interventions to address the losses discussed and fostering community support, all of which can enhance pain management and psychological well‐being. These actionable steps could help to address the overwhelming impact of pain in these women's lives and support them to live well with pain, providing the validation, support and resources they need to feel empowered. By doing so, we can begin to restore dignity and agency to the many women living with pain, creating a future where their voices are heard and their needs are met with compassion and action.

## AUTHOR CONTRIBUTIONS

**Sukhvinder Biring:** Conceptualization; data curation; formal analysis; investigation; methodology; project administration; writing – original draft; writing – review and editing. **Amy E. Burton:** Conceptualization; methodology; supervision; writing – original draft; writing – review and editing. **Lynn Dunwoody:** Conceptualization; methodology; writing – review and editing; writing – original draft. **Peter Kevern:** Conceptualization; data curation; formal analysis; investigation; methodology; writing – original draft; writing – review and editing.

## CONFLICT OF INTEREST STATEMENT

The authors declare no conflict of interest.

## ETHICS STATEMENT

Ethical approval is not required for this study, however a disclaimer was submitted and approved by Staffordshire University Psychology departmental ethics committee. The review protocol was registered in the International Prospective Register of Systematic Reviews (registration number: CRD42022331582).

## Data Availability

Data is available from the corresponding author by request.
